# The genome of *Shorea leprosula* (Dipterocarpaceae) highlights the ecological relevance of drought in aseasonal tropical rainforests

**DOI:** 10.1038/s42003-021-02682-1

**Published:** 2021-10-07

**Authors:** Kevin Kit Siong Ng, Masaki J. Kobayashi, Jeffrey A. Fawcett, Masaomi Hatakeyama, Timothy Paape, Chin Hong Ng, Choon Cheng Ang, Lee Hong Tnah, Chai Ting Lee, Tomoaki Nishiyama, Jun Sese, Michael J. O’Brien, Dario Copetti, Mohd Noor Mat Isa, Robert Cyril Ong, Mahardika Putra, Iskandar Z. Siregar, Sapto Indrioko, Yoshiko Kosugi, Ayako Izuno, Yuji Isagi, Soon Leong Lee, Kentaro K. Shimizu

**Affiliations:** 1 Department of Evolutionary Biology and Environmental Studies, University of Zurich, Zurich, Switzerland; 2 Genetics Laboratory, Forest Research Institute Malaysia (FRIM), Kepong, Selangor Malaysia; 3 URPP Global Change and Biodiversity, University of Zurich, Zurich, Switzerland; 4 Forestry Division, Japan International Research Center for Agricultural Sciences (JIRCAS), Tsukuba, Ibaraki Japan; 5 Artificial Intelligence Research Center, National Institute of Advanced Industrial Science and Technology (AIST), Tokyo, Japan; 6 Department of Evolutionary Studies of Biosystems, SOKENDAI, The Graduate University for Advanced Studies, Hayama, Kanagawa Japan; 7 RIKEN iTHEMS, Wako, Saitama, Japan; 8 Functional Genomics Center Zurich, Zurich, Switzerland; 9 Swiss Institute of Bioinformatics (SIB), Lausanne, Switzerland; 10 Division of Integrated Omics research, Research Center for Experimental Modeling of Human Disease, Kanazawa University, Kanazawa, Japan; 11 AIST-Tokyo Tech RWBC-OIL, Meguro-ku, Tokyo, Japan; 12 Humanome Lab Inc., Chuo-ku, Tokyo, Japan; 13 Área de Biodiversidad y Conservación, Universidad Rey Juan Carlos, c/Tulipán s/n., E-28933 Móstoles, Spain; 14 Molecular Plant Breeding, Institute of Agricultural Sciences, ETH Zurich, Zurich, Switzerland; 15 Malaysia Genome Institute, Kajang, Selangor Malaysia; 16 Forest Research Centre, Sandakan, Sabah Malaysia; 17 Faculty of Forestry, Bogor Agricultural University, Bogor, Indonesia; 18 Faculty of Forestry, Gadjah Mada University, Yogyakarta, Indonesia; 19 Graduate School of Agriculture, Kyoto University, Kyoto, Japan; 20 Forestry and Forest Products Research Institute (FFPRI), Tsukuba, Ibaraki Japan; 21 Kihara Institute for Biological Research, Yokohama City University, Yokohama, Japan

**Keywords:** Tropical ecology, Genome duplication, Forest ecology, Forestry

## Abstract

Hyperdiverse tropical rainforests, such as the aseasonal forests in Southeast Asia, are supported by high annual rainfall. Its canopy is dominated by the species-rich tree family of Dipterocarpaceae (Asian dipterocarps), which has both ecological (e.g., supports flora and fauna) and economical (e.g., timber production) importance. Recent ecological studies suggested that rare irregular drought events may be an environmental stress and signal for the tropical trees. We assembled the genome of a widespread but near threatened dipterocarp, *Shorea leprosula*, and analyzed the transcriptome sequences of ten dipterocarp species representing seven genera. Comparative genomic and molecular dating analyses suggested a whole-genome duplication close to the Cretaceous-Paleogene extinction event followed by the diversification of major dipterocarp lineages (i.e. Dipterocarpoideae). Interestingly, the retained duplicated genes were enriched for genes upregulated by no-irrigation treatment. These findings provide molecular support for the relevance of drought for tropical trees despite the lack of an annual dry season.

## Abstract (web summary)

Ng, Kobayashi, et al. present the genome assembly for the dipterocarp *Shorea leprosula* and present transcriptome data for ten more dipterocarp species of seven genera. The *Shorea leprosula* genome, along with drought stress experiments, demonstrate evidence for a whole-genome duplication (WGD) event dated to the Cretaceous-Paleogene boundary, with WGD duplicates enriched in genes upregulated during the drought response.

## Introduction

Average annual rainfall is the highest in tropical rainforests, which harbor hotspots of biodiversity. Southeast Asian tropical rainforests are commonly aseasonal, without distinct intra-annual dry seasons, and are characterized by the dominant canopy tree family of Dipterocarpaceae^1–3^. Recent research has pursued the importance of rainfall variation and drought for promoting species distribution^4^ and for triggering reproduction^5–8^ in tropical forests, although ecologists have long-viewed light and soil characteristics as the main drivers of environmental filtering and species distributions in ever-wet tropical forests^9^. Drought events in this system are often associated with irregular supra-annual El Niño Southern Oscillations (ENSO), and climate models project more frequent and severe ENSO events^10–12^. These increased drought patterns could alter synchronous general flowering^5–8,13^, reduce plant growth and carbon sequestration^14^, increase tree mortality^15,16^, and shift species composition^17^.

To complement the existing ecological studies, genomic studies may elucidate the potential importance of the inter-annual drought on plants. One of the major limitations of tropical plant studies is the paucity of genetic and genomic data for species of environmental and forestry relevance in contrast to crop and commodity-producing species (cacao^18^, rubber tree^19^, oil palm^20^, and durian^21^). Nonetheless, several molecular studies using real-time PCR or de novo transcriptome approaches of Dipterocarpaceae suggested that expression levels of phenology- and stress-related genes^7,8^ were associated with ENSO-related fluctuations in drought or temperature. This premises that a genome assembly would be valuable to test the relevance of drought in tropical trees.

The dominant tree family, Dipterocarpaceae (comprised of >500 species) has the center of diversity in tropical Southeast Asia, where 488 species of the subfamily Dipterocarpoideae are found^1,2^. Their evolutionary origin remains enigmatic. While many dipterocarp researchers have proposed an ancient origin of the family in Gondwanaland (e.g., >120 Ma (million years ago))^3^, molecular dating studies have suggested a much younger date^22,23^. In support of the importance of inter-annual drought events, dipterocarp species appear to have maintained a functional response to drought at the community level, which promotes species coexistence^24^ and diversity^25^ and synchronizes reproduction^5–8^. Besides their ecological importance, Asian dipterocarps lead the international tropical timber market, therefore playing an important role in the economy of many countries within the region^26^. They are critically important as keystone species and serve as active carbon sink^3^. Despite the research activities of Asian dipterocarps dating back to 1825^27^, the main issue in tropical tree breeding and improvement are the complexity and cost of the breeding programs as well as the long breeding cycles. Additionally, many of the dipterocarp species are now categorized as near threatened or endangered as a result of exploitation and massive population reduction^28^, further indicating the need of the genomic resources for strengthening research related to genetic conservation of dipterocarps^29,30^.

Here, we report a draft genome assembly of *Shorea leprosula*, a species that has been used as a representative of Dipterocarpaceae to assess genetic diversity by allozymes, nuclear SSR, AFLPs, and chloroplast loci^31–35^. It is locally known as Meranti Tembaga, and is internationally traded under the Light Red Meranti timber group. This species is widely distributed throughout aseasonal tropical rainforests of Southeast Asia (Peninsular Malaysia, Borneo, and Sumatra)^1,36^, but is classified as a near-threatened category under the IUCN Red List^37^. We showed that an ancient whole-genome duplication (WGD) event coincided with the Cretaceous–Paleogene (K-Pg) boundary using the genome-wide data of 19 distribution-wide *S. leprosula* individuals as well as of 10 species from seven genera of Dipterocarpaceae. Genes that were upregulated by no-irrigation treatment were significantly enriched in the retained duplicated genes. Climate data supported that *S. leprosula* is distributed in the environments with irregular drought despite the lack of annual dry season. The availability of the genome assembly of a dipterocarp is of great utility for genetic conservation and plant breeding in facing global changes.

## Results

### Genome assembly

**Fig. 1:**
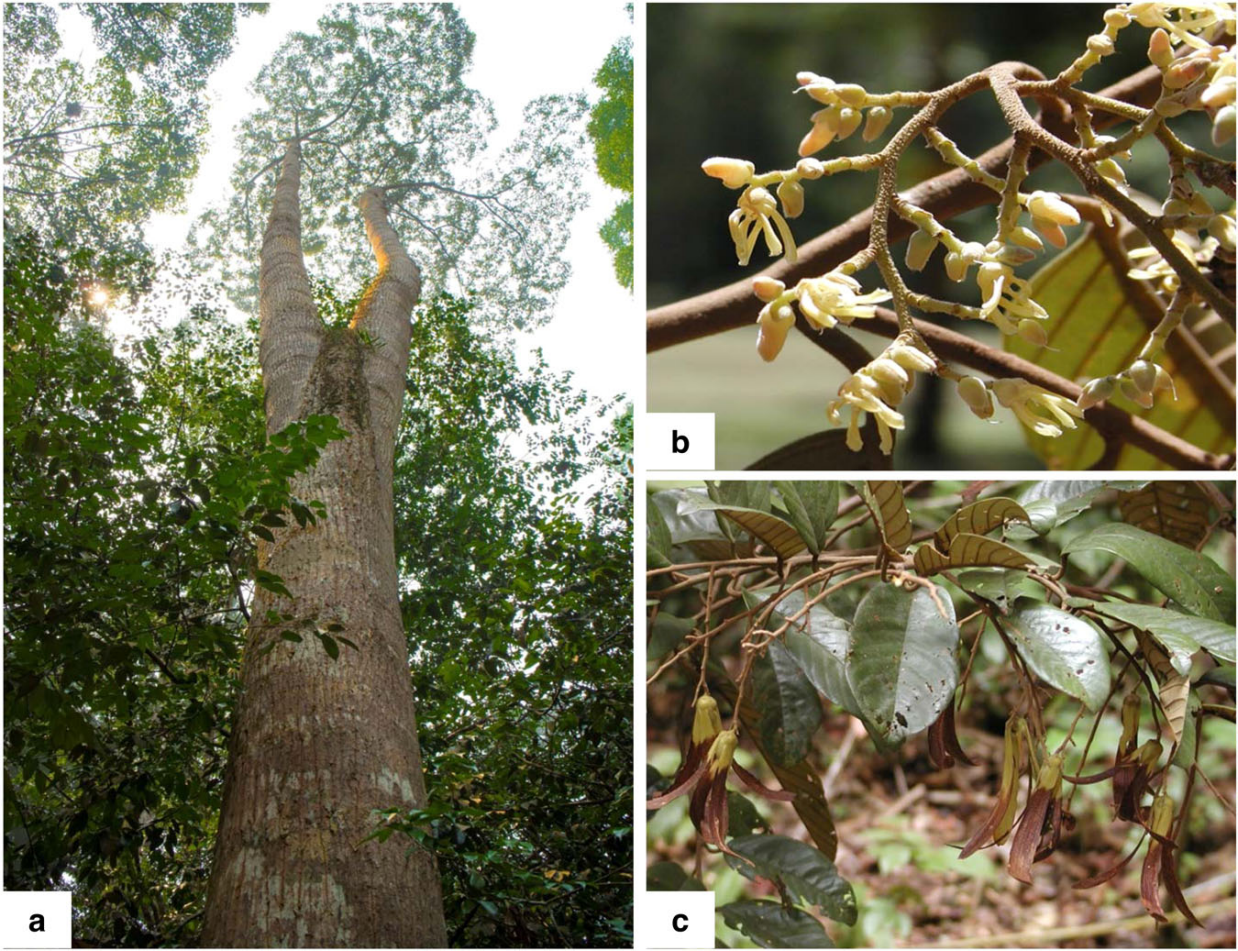
The *Shorea leprosula* tree that was used for genome sequencing. **a** Tree trunk. **b** Flowers. **c** Mature winged fruits.

Whole-genome sequencing of *S. leprosula* (Fig. 1) was performed on Illumina HiSeq platform, using paired-end and mate-pair libraries with various insert sizes ranging from 170 bp to 17 kb, with over 380-fold coverage of its haploid genome (*n* = 7)^38,39^ (Supplementary Table 1). The contig and scaffold N50 lengths obtained from the ALLPATHSLG^40^ assembly were 7.8 kb (spanning the longest 71,752 contigs) and 2.07 Mb (with 2913 scaffolds above 1 kb), respectively. The total size of the assembly of scaffolds was 340.5 Mb (Table 1). Thus, the scaffolds covered ~85% and ~87% of the estimated genome ∼402 Mb by flow cytometry^41^ and ~391 Mb by k-mer distribution^42^, respectively. K-mer Analysis Toolkit (KAT)^43^ analysis revealed two peaks (Supplementary Fig. 1), confirming the genome of *S. leprosula* is heterozygous. The frequency of the k-mers in the assembly confirmed that the assembly is haploid (i.e., only one of the two heterozygous variants is present).

**Table 1:** Summary statistics of the *Shorea leprosula* draft genome assembly.

Assembly features	Statistics
Estimated genome size	402 Mb (by flow cytometry) 391 Mb (by k-mer distribution)
Number of scaffolds	2913
Scaffold N50 excluding gaps	2.07 Mb
Scaffold N50 including gaps	2.58 Mb
Longest scaffold excluding gaps	8.15 Mb
Number of contigs	71,752
Contig N50	7.80 kb
Assembly length excluding gaps	340.50 Mb
Assembly length including gaps	449.70 Mb
Transposable elements and repeat region percentage of assembly	32.80%
Predicted gene models	60,563
Gene length (amino acids)	Mean: 350.28; median: 246
Annotated gene models (*Theobroma cacao/Arabidopsis thaliana* gene models)	43,868
Unannotated gene models	16,695

To validate the genome assembly, we mapped all paired-end and mate-pair reads to the assembled genome and found that the vast majority of the reads (93.35%) aligned (Supplementary Table 1). To assess the completeness of our assembly, we compared it to 1440 core genes in the Embryophyta lineage using BUSCO^44^, finding that 93.3% of them were present (79.7% in a single copy, 13.6% in two copies), with only 2.5% and 4.2% fragmented or missing, respectively, comparable to available assemblies of cacao (95.8%)^18^ and durian (90.3%)^21^ in Malvales. We also confirmed that the vast majority of RNA-seq reads of seven organs of *S. leprosula* (namely leaf buds, flower bud, flower, inner bark, small seed, large seed, and calyx) obtained from the sequenced individual were mapped on the assembly (~86%) (Supplementary Table 2).

### Genome annotation

To annotate the *S. leprosula* assembly, we first identified transposable elements and non-genic repeated sequences. We found that about 132 Mb of sequence (corresponding to 33% of the assembly) were attributed to transposable elements and repeats (Table 1 and Supplementary Table 3). Gene prediction with AUGUSTUS^45^ and the RNA-seq reads of seven organs described above resulted in 60,563 protein-coding gene models (Supplementary Table 4). In a further evaluation, the *S. leprosula* models were compared with the protein-coding genes of *Theobroma cacao*^18^ (cacao, Malvaceae, which is distantly related in Malvales and is still the closest well-characterized relative of Dipterocarpaceae without lineage-specific genome duplication) and *Arabidopsis thaliana*, and we found that 43,868 genes were supported by homology. Moreover, out of the 43,868 genes with homology, 20,690 genes showed synteny with the *T. cacao* assembly by using MCScanX. Based on these empirical supports, we classified the predicted genes into three categories: category A for the 20,690 genes with synteny; category B for the 23,178 genes with homology with either *T. cacao* and/or *A. thaliana* but without synteny; category C for the 16,695 genes without clear homology (Supplementary Fig. 2 and Supplementary Tables 4 and 5). Category C was composed mostly of predicted genes shorter than 80 or 50 amino acids (aa) (mean length ~122 aa compared to those in the categories A and B being 414 and 458 aa on average, respectively (Supplementary Table 5).

To test whether genes in the A and B categories are also present in the individuals from different populations and other dipterocarp species, we analyzed the resequencing data of 19 *S. leprosula* individuals covering the distribution range (Borneo, Sumatra, and Peninsular Malaysia, Supplementary Table 6), obtaining 673,772 SNPs. The resequencing of three dipterocarp species *Shorea platycarpa, Neobalanocarpus heimii*, and *Dryobalanops aromatica* (Supplementary Table 7) showed relatively high mapping rate (73–92%), allowing the identification of homologs. We found that 30,677 (70%) out of 43,868 genes of categories A and B were present in all the studied individuals and species (Supplementary Table 5). Using the 30,677 genes that were found in all samples (Supplementary Table 4), genome-wide average nucleotide diversity (π), Watterson’s theta (*θ*_w_), and Tajima’s *D* values were estimated as 0.0072, 0.0095, and −0.9801, respectively (Supplementary Table 8 and Supplementary Fig. 3), which was comparable to a previous study that used fewer nuclear loci^46^. Admixture analysis of 19 individuals of *S. leprosula* from Peninsular Malaysia, Sumatra, and Borneo based on the cross-validation error plot suggests the presence of two subpopulations (*K* = 2) (Supplementary Fig. 4); where the samples from Borneo were split from those of Peninsular Malaysia and Sumatra (Supplementary Fig. 5). Because of the empirical support in closely related species and populations, and longer protein sequences, we considered that the gene models in the categories A and B (43,868 genes) were of high-confidence genes.

### Ancient whole-genome duplication (WGD)

**Fig. 2:**
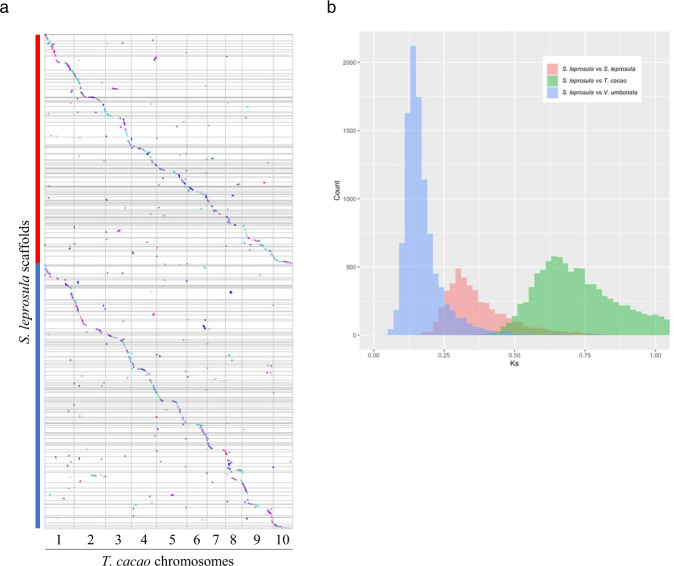
Assessment of whole-genome duplication. **a** Collinearity dotplot between *Theobroma cacao* chromosomes and *Shorea leprosula* scaffolds. Dots with different colors represent different collinear blocks. Chromosomes and scaffolds sequence are separated by gray line. Source data on the order and the orientation of the *S. leprosula* scaffolds used for the dotplot are found in Supplementary Table 9. Dotplot was generated based on the results of MCScanX using VGCS2.0. Red and blue lines correspond to the two sets of the *S. leprosula* scaffolds (set 1 and 2 in Supplementary Table 9). **b** Ks distribution of *S. leprosula* paralogs in collinear blocks (*n* = 4513), orthologs of *S. leprosula* and *T. cacao* (*n* = 11,239), and orthologs of *S. leprosula and Vatica umbonata* (*n* = 10,280) are shown in red, green, and blue, respectively. Note that the average ratio of the Ks of the *S. leprosula*–*T. cacao* orthologs and the Ks of the *V. umbonata*–*T. cacao* orthologs was 1.00, suggesting that the rates of synonymous substitutions in *S. leprosula* and *V. umbonata* are highly similar. Source data are provided as Supplementary Data 1. The Ks distribution of the orthologs of *S. leprosula* and the remaining Dipterocarpoideae species are shown in Supplementary Fig. S6.

In order to understand the genome evolution in Dipterocarpaceae, we assessed synteny between *S. leprosula* and *T. cacao*. As visualized in a dotplot (Fig. 2a and Supplementary Table 9), most *T. cacao* genomic regions were syntenic to two genomic regions of *S. leprosula*. This suggested that the entire genome of *S. leprosula* duplicated after its divergence from the lineage of *T. cacao*. Among the 20,690 *S. leprosula* genes (category A) that had syntenic homologs in *T. cacao*, more than half (12,886 genes, 62%) were retained as duplicates in the collinear blocks of *S. leprosula* (Supplementary Tables 4 and 10). We then estimated the expected number of synonymous substitutions per synonymous site (Ks) among the *S. leprosula* collinear duplicates. The Ks distribution showed a single and distinct peak around Ks = 0.3 (Fig. 2b). This result further supports that these genes duplicated around the same time, most probably via a single WGD event. In addition, the Ks estimates between the *S. leprosula* and *T. cacao* orthologs were considerably larger than those between the *S. leprosula* collinear duplicates (hereafter, referred to as “the WGD-retained duplicates”) (Fig. 2b). This is consistent with the WGD being specific to the lineage of *S. leprosula* and not shared with the lineage of *T. cacao*, and also suggests that the WGD is considerably younger than the divergence of Dipterocarpaceae and Malvaceae.

To test whether the WGD can be observed in (is shared with) the other dipterocarp species, we examined the Ks distributions of the duplicated genes (between 4004 to 7108 genes) obtained by transcriptome assembly of 10 other species from seven different genera (Supplementary Tables 4 and 11). The Ks distributions of all species also had single peaks around Ks = 0.3 (Supplementary Fig. 6a), suggesting that the WGD event occurred before the split of the examined species in Dipterocarpoideae. To validate this finding further, we also checked the Ks distributions of ortholog pairs between *S. leprosula* and the other Dipterocarpoideae species (Fig. 2b, Supplementary Fig. 6b, and Supplementary Data 1). In all the studied species, the peak of Ks estimates for orthologous genes was lower than the peak corresponding to the WGDs. Taken together, these results place the WGD event after the split from *T. cacao*, but before the divergence of the examined Dipterocarpoideae species.

### The WGD event coincided with the K-Pg boundary, as in other plant lineages

**Fig. 3:**
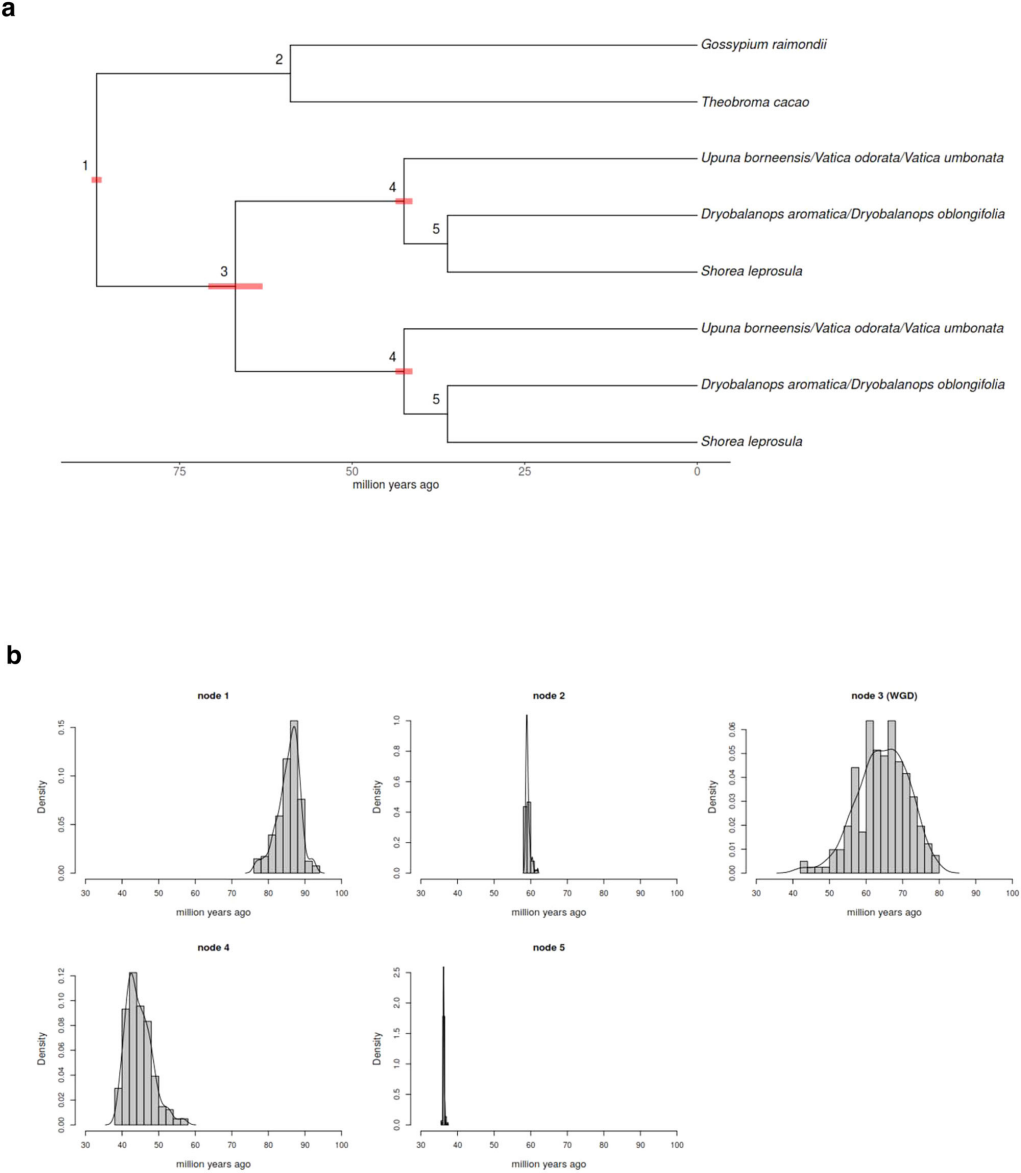
Time estimation of the whole-genome duplication. **a** Representative gene tree used for phylogenetic dating and the estimated ages of each node, which are the modes of the kernel density estimates of the age distributions shown in (**b**), based on the parameter Setting 1. The red bars correspond to the 95% confidence intervals that were obtained by calculating the mode of 1000 bootstrap density estimates of the ages of each family shown in (**b**). Only the confidence intervals with a range of >1 million years are shown. Note that these are not posterior uncertainty intervals and does not take into account the posterior uncertainty in each individual family (see Supplementary Table S12 for the high posterior density of each family). Node 3 corresponds to the dipterocarp WGD. Source data are shown in Supplementary Table S13. **b** Age distribution of the divergence of the nodes based on the parameter Setting 1. Source data are shown in Supplementary Table S12.

To further understand when the WGD event occurred, we estimated the timing of the WGD event by focusing on the WGD-retained duplicates in *S. leprosula* that have syntenic homologs in the *T. cacao* genome. To obtain an age estimate of the WGD, phylogenetic dating was performed (Supplementary Data 2) using a Bayesian evolutionary analysis framework previously described^47^ for 204 orthologous groups with cleaned alignment lengths of at least 100 aa. For each of these orthologous groups, the dates for each node were estimated by incorporating fossil calibrations and the dates obtained from previous studies (i.e., secondary calibrations) as prior information to account for the uncertainty in the ages of the calibrations. Using two different calibration settings (Supplementary Table 12), we estimated the timing of the WGD event as 66.9 Ma (95% CI, 61.3–69.3 Ma) and 69.7 Ma (95% CI, 67.7–75.3 Ma). Likewise, the divergence between the Dipterocarpaceae and Malvaceae was estimated to be ~86–98 Ma, whereas the divergence between the different dipterocarp lineages represented by the nodes 4 and 5 were estimated to be ~42–50 and ~36–40 Ma, respectively (Fig. 3, Supplementary Figs. 7–9 and, Supplementary Table 13). These results suggest that the ancestral dipterocarp lineage underwent a WGD close to the Cretaceous–Paleogene (K-Pg) extinction event of ~66 Ma, as in many other angiosperm plant linegaes^47,48^.

### Characterization of duplicated genes in dipterocarps

We next characterized the WGD-retained duplicates in the *S. leprosula* genome. First, we focused on their overall evolutionary trends. Previous studies suggest that genes retained as duplicates after WGD tend to show slower evolutionary rates at nonsynonymous sites than genes not retained as duplicates during the long rediploidization processes (loss of some gene duplicates after WGD)^49,50^. To test whether a similar trend is observed in the WGD-retained duplicates of *S. leprosula*, we estimated Ka, Ks, and Ka/Ks using the orthologs between *S. leprosula* and *T. cacao* and compared the results between the WGD-retained duplicates and the genes that lost the syntenic duplicates derived from the WGD event (“the non-retained genes”). Our analysis showed that Ka and Ka/Ks estimates for the WGD-retained duplicates were significantly lower than those for the non-retained genes (Supplementary Fig. 10), indicating slower evolutionary rates of the WGD-retained duplicates at nonsynonymous sites. In contrast, such a significant difference was not observed for the Ks estimates between the WGD-retained duplicates and the non-retained genes (Supplementary Fig. 10). Therefore, changes in substitution rates were specific to the nonsynonymous sites.

Gene retention and loss are shown to be nonrandom with respect to gene function in ancient polyploids^51–56^. Hence, we examined the common functions of the 12,886 WGD-retained duplicates using a gene ontology (GO) enrichment test against the GO terms of *A. thaliana* orthologs. A large number of genes related to transcriptional regulation, signal transduction, and development were retained (Supplementary Table 14), consistent with previous findings reported for *A. thaliana* and other plants^51–55^. In addition to these terms commonly enriched in retained duplicated genes, drought-related terms, such as “response to salt stress” and “response to abscisic acid” were also found. To test whether the retention of the drought-related genes is specific to *S. leprosula* or a common feature among the Dipterocarpoideae species, we investigated the retention of these duplicated genes in the resequencing data obtained from the population and the interspecific samples (Supplementary Tables 6 and 7). Of the 12,886 WGD-retained duplicates, most of them (87%, 11,250 genes) had both copies of the corresponding homologs (Supplementary Table 15). A GO enrichment test of this conserved gene set also yielded similar results including “response to abscisic acid” (Supplementary Table 16). These data suggest that the retention of the drought-related duplicated genes is a common feature among the Dipterocarpoideae species in aseasonal tropics, rather than being a lineage-specific character of *S. leprosula*.

We also examined the common functions of tandemly duplicated genes in the *S. leprosula* genome by GO enrichment test. We found that 1212 genes in the category A had tandemly duplicated copies (Supplementary Table 4), and that their enriched functions were not overlapped with those of the WGD-retained duplicates (Supplementary Table 17).

### Functional analysis of drought-responsive genes via no-irrigation treatment

**Fig. 4:**
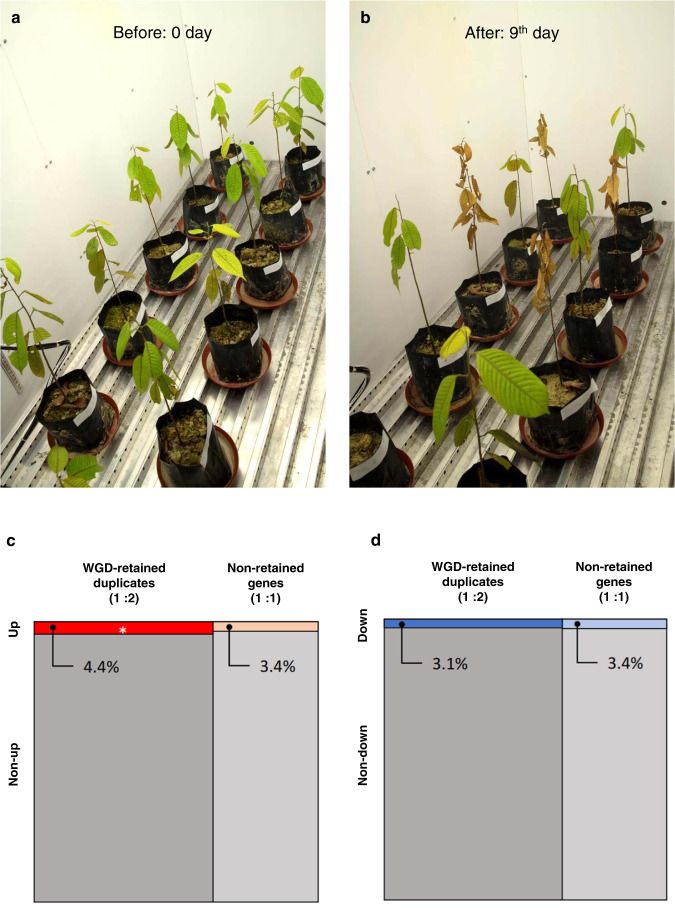
No-irrigation treatment on *Shorea leprosula* seedlings carried out in a growth chamber. **a** *S. leprosula* seedlings at 0th day of treatment. **b** On the 9th day of the treatment. Seedlings with no-irrigation treatment had brown withered leaves, while the control seedlings with 50 mL of water daily had green leaves. **c**, **d** Mosaic plots to check enrichment of upregulated (**c**) and downregulated (**d**) drought-response genes in the *S. leprosula* WGD-retained duplicates. An asterisk in (**c**) shows significant enrichment of the upregulated genes in the *S. leprosula* WGD-retained duplicates (*P*-value after Bonferroni correction: 0.0004). Up: upregulated genes, Non-up: non-upregulated genes. The source data are shown in Table 2.

Although we obtained results showing that drought-related genes were significantly enriched in the WGD-retained duplicates using the GO terms assigned based on the homologies to the *A. thaliana* orthologs, homology to functionally verified *A. thaliana* genes does not ensure that the *S. leprosula* homologs also have a role in response to drought. Therefore, we characterized drought-responsive genes of *S. leprosula* by performing a no-irrigation treatment of *S. leprosula* seedlings (Supplementary Table 18). Leaf samples were collected for RNA-seq analysis at the beginning of the treatment and at the 7th day, which was slightly before the 9th day when the typical wilting symptom (withered and brown leaves) was observed (Fig. 4a, b). Under this water stress condition, we conducted an expression analysis using genes from all three categories. Differential expression analysis identified 1200 upregulated and 914 downregulated genes in total, of which the A category had 829 and 658 genes, respectively (Supplementary Fig. 11 and Supplementary Tables 19 and 20). In the upregulated gene list, the highest-ranking GO terms were similar to those known to be involved in the drought response, such as “response to water deprivation”, “response to abscisic acid”, and “response to salt stress” (Supplementary Table 21**)**. In addition, the enriched categories encompassed “response to chitin” and “response to oxidative stress”, which may be attributable to the crosstalk of the signaling of abscisic acid, wounding, and defense facing the high pressure of pathogens in the tropics^57,58^. GO terms related to photosynthesis, light, and biosynthetic processes (starch, chlorophyll, glycogen, and amylopectin) were enriched among the downregulated genes (Supplementary Table 22).

Using the genes that responded to the no-irrigation treatment, we tested whether these are significantly enriched in the WGD-retained duplicates. Fisher’s exact test showed significant enrichment of upregulated genes in the WGD-retained duplicates (Bonferroni corrected *P* = 0.0004, Table 2, and Fig. 4c), in contrast to non-significant enrichment for the downregulated genes (Bonferroni corrected *P* = 1.0000, Table 2 and Fig. 4d). This result is consistent with that obtained in the GO analysis described above, and indicates that the observed enrichment of drought-response genes is not likely due to artifacts in the GO enrichment test based on the homologies to the *A. thaliana* orthologs. These WGD-retained drought-up genes also showed slower evolutionary rates at nonsynonymous sites, compared with the non-retained genes (Supplementary Fig. 10). On the other hand, such significant enrichments of drought-response genes were not found in the tandemly duplicated genes (Supplementary Table 23), similarly to the results of the GO enrichment test (Supplementary Table 17). We found that the list of WGD-retained drought-up genes encompassed genes involved in diverse molecular roles in drought stress-response pathways (Supplementary Table 19), including a homolog of *ABI1* (encoding a receptor component of plant hormone abscisic acid), *DREB2C* (encoding a key transcriptional factor in dry treatment), and *TIP1* and *TIP3* (encoding water-transport aquaporins) (Supplementary Fig. 11). These results support the hypothesis that some of the WGD-retained duplicates in the Dipterocarpoideae species tend to function in drought response.

**Table 2:** Comparisons between the WGD-retained genes and the differentially expressed genes under the no-irrigation treatment.

Genes	WGD-retained and differentially expressed	WGD-retained and not differentially expressed	Non-retained and differentially expressed	Non-retained and not differentially expressed	*P*-value after Bonferroni correction
Downregulated genes (FDR < 0.05)	402	12,484	256	7338	1.0000
Upregulated genes (FDR < 0.05)	570	12,316	259	7335	0.0004

### Irregular drought instead of annual dry season

To examine whether the populations of *S. leprosula* experience dry environments, we analyzed multiple datasets of precipitation across the range of *S. leprosula*. First, we extracted the precipitation of the driest month across the spatial range of *S. leprosula* from the WorldClim data. Despite a broad variation, most localities (173) across the range of *S. leprosula* had greater than 100 mm of rainfall in the driest month (i.e., the driest month still met the evapotranspiration demands at the site). These values showed little overlap with species in seasonal forests (*Shorea roxburghii* is provided as an example from seasonal forests; Supplementary Fig. 12a, b). These data indicate few sites across the range of *S. leprosula* have an annual dry season. Second, we analyzed average 30-day cumulative rainfall from 2001 to 2014 measured at two localities within the distribution of *S. leprosula* (Pasoh Forest Reserve, Peninsular Malaysia, and Danum Valley Field Centre, Borneo). We found it fell below 100 mm roughly 20% in Pasoh and roughly 5% in Danum site (Supplementary Fig. 12c–f). The latter site was wetter but there were still supra-annual drought events (below 100 mm in 2002 and 2010). The combination of these modeled and observed climate data suggest that *S. leprosula* is distributed in the environments with irregular drought events even if they lack annual dry season.

## Discussion

We sequenced the genome of *S. leprosula* using Illumina paired-end and mate-pair sequencing strategy, yielding sequence dataset of ~388-fold genome coverage. K-mer analysis, BUSCO analysis, and high-read-mapping rate indicated the completeness and accuracy of our genome assembly. We annotated 43,868 high-confidence genes showing homology to *T. cacao* and *A. thaliana* proteomes.

Our comparative genomic and molecular dating results, together with many recent studies on angiosperm evolution, allow us to propose the following scenario regarding the evolution and biogeography of Dipterocarpaceae. First, the Dipterocarpaceae lineage split from the lineage of Malvaceae in the Late Cretaceous, followed by a WGD in the common ancestor of the Dipterocarpoideae species close to the K-Pg boundary, after which the Dipterocarpoideae lineages diverged during the Eocene. Thus, Dipterocarpaceae provides another example that has been observed across many plant groups where the diversification occurred following a WGD around the K-Pg extinction event^59–61^. This timeline contrasts with the scenario hypothesized by many dipterocarp researchers which posits that the Dipterocarpaceae originated on Gondwanaland >120 Ma^3^ or >135 Ma^62^, and that Dipterocarpaceae and related lineages distributed in South America, Africa, Madagascar, Seychelles, and Asia diverged due to the breakup of the Gondwanan landmasses^3,63^, i.e., Gondwanan vicariance. However, the Gondwanan origin of Dipterocarpaceae is clearly not consistent with the generally accepted timeline of the divergence of various angiosperm/eudicot clades, in particular, that the origin of the eudicots should not be much older than ∼130 Ma^22,64^. Moreover, molecular dating studies of many different Tropical and Southern Hemisphere plant groups that show “Gondwanan” trans-continental distributions have reported much younger divergence date estimates that are not consistent with a strict Gondwanan vicariance scenario^65–69^. Our estimates of the divergence between Dipterocarpaceae and Malvaceae (86–98 Ma) are much younger than the proposed dates assuming a Gondwanan origin, as expected considering our priors, although it is worth noting that they are slightly older than previous estimates based on molecular dating (~70–80 Ma^22,63^). In addition, we obtained much younger estimates for the divergence of the Dipterocarpoideae lineages that are not consistent with the assumption that the separation of India and the Seychelles caused the divergence of certain lineages (see also “Methods”)^3,63^.

The main hypothesis to explain the inconsistencies between the molecular dating results and vicariance scenarios in many plant lineages is that long-distance and trans-oceanic dispersals are much more common than thought before^65–69^. For Dipterocarpaceae, such dispersals have been considered highly unlikely because their seeds lack dormancy, show salt-intolerance, and have low dispersal capacity^3,63^. Yet, results indicating long-distance dispersal have also been obtained for plant groups, such as the *Nothofagus* species, which show trans-oceanic distributions despite having poor dispersal capacity^65^. It is also worth noting that the exact timing and nature of the Gondwanan breakup is debatable, and that there may have been connected landmasses that enabled overland dispersal after the various proposed dates of the separation of landmasses^66,67,70^. Thus, while our results, combined with various recent findings, suggest that dispersal played a key role in the trans-continental distribution of Dipterocarpaceae, its exact mechanism remains an open question that is relevant also to many other plant groups.

It is known that the aseasonal tropical rainforests of Southeast Asia region (where dipterocarps dominate) receives high annual rainfalls. *S. leprosula* is a typical species in aseasonal tropical rainforests, and the precipitation of the driest month in its habitat is clearly higher than those in the habitat of *S. roxburghii*, which is a species in seasonal tropics (Supplementary Fig. 12a, b). Although *S. leprosula* inhabits regions with no annual dry season, our results showed that the drought-up genes are preferentially retained after the WGD event in this species (Table 2 and Fig. 4), and these WGD-retained drought-up genes are likely to conserve their functions because of their slower evolutionary rates at nonsynonymous sites (Supplementary Fig. 10). It is yet to be shown whether these substitution rate differences are biologically relevant. Nevertheless, the WGD-retained duplicates were conserved among the three species in different genera (*Shorea*, *Dryobalanops*, and *Neobalanocarpus*) inhabiting aseasonal tropics as well as among the 19 *S. leprosula* individuals of different populations (Supplementary Tables 15 and 16). The observed conservation suggests that these WGD-retained drought-related genes have been functionally important, not only at the WGD event, but also during the subsequent period in dipterocarp species in aseasonal tropics. At the WGD event, the genome duplication and duplicated drought-related genes might allow the ancestral dipterocarp species to develop tolerance to harsh environments during the mass-extinction period of the K-Pg boundary because contemporary polyploids often show enhanced environmental tolerance^71–73^. After the period around the WGD event, paleoclimate studies suggest that Asian dipterocarps lived in climates with dry seasons^74–76^, which might have contributed to the retention of the WGD-derived drought-related genes. In the present-day condition, aseasonal tropics in Southeast Asia receive high annual rainfalls and also suffers from occasional drought mostly due to ENSO. Although such drought conditions rarely occur, the irregular supra-annual drought (Supplementary Fig. 12) may be the basis for the preferential retention of drought-related duplicated genes in the Asian dipterocarps of aseasonal tropics. The observed preferential retention of the WGD-derived drought-related genes does not contradict the recent ecological studies that showed the relevance of inter-annual drought events in dipterocarp species in aseasonal tropical rainforests in Southeast Asia^5–8,24,25^. Nevertheless, it is still difficult to reveal the significance of an additional copy of a drought-related gene. We note that the enrichment of retained drought-related genes in Dipterocarpaceae was originated by WGD (Supplementary Tables 14 and 16) rather than by tandem duplication (Supplementary Table 17), in contrast to lineage-specific tandem duplication of stress-related genes reported in e.g., *A. thaliana*^77^.

In 2015, Malaysia and Indonesia contributed over 37.8% (93.7 million m^3^) of the total global production of tropical saw and veneer log, and more than 70% (4.8 million m^3^) of the total global export of plywood^26^. The growing demand for timber and timber products requires that tree breeders accelerate the improvement of germplasm. The lack of improved planting materials and knowledge of genetic and genomic resources such as the availability of high-density markers or even genetic maps for any dipterocarps hinders the success of forestry plantation. Our data of genome assembly, genome-wide polymorphisms, and divergence between 10 additional dipterocarp species will serve as a solid basis for establishing a molecular breeding program for Dipterocarpaceae. Here, we identified 673,772 SNPs by the resequencing of 19 individuals throughout the distribution range. The population structure analysis showed the split of Bornean populations from those of Peninsular Malaysia and Sumatra, which informs the design of breeding and association studies. Our findings support the hypothesis stating that canopy trees^35,78,79^ and other terrestrial organisms^80–82^ in Sundaland were divided into two clusters from the drowning of Sunda Shelf after the Last Glacial Maximum^83^.

Dipterocarp species are keystones in Asian tropical ecosystems. The biomass estimates of natural Asian dipterocarp forests range from 205 to 496 Mg per ha^84–86^, with biomass values 30–60% higher than those of the corresponding forest in Amazonia^87–89^, which highlights their high carbon storage value^3^. Presently, a large number of dipterocarp species have and are currently being planted and monitored in the Sabah Biodiversity Experiment and FRIM’s Common Garden Experiment sites, and thus would provide opportunities for establishing genome-wide association studies, genomic selection, and ecological genomics analyses^29,30^. Considering the critical contribution of tropical forests to the earth systems, it is urgent to fill the gap of molecular knowledge about tropical trees to a level that is comparable to that of temperate regions.

## Methods

### Sequencing of *Shorea leprosula* genome

#### Sample collection

Leaf samples of *S.* *leprosula* were obtained from a reproductively mature (diameter at breast height, 50 cm) diploid tree B1_19 (DNA ID 214) grown in the Dipterocarp Arboretum, Forest Research Institute Malaysia (FRIM).

#### DNA extraction

Genomic DNA was extracted from leaf samples using the 2% cetyltrimethylammonium bromide (CTAB) method^90^ and purified using a High Pure PCR Template Purification kit (Roche).

#### Library preparation and sequencing

Paired-end (170, 500, and 800 bp) and mate-pair (2 kb) genomic libraries were prepared using a TruSeq DNA Library Preparation kit (Illumina) and a Mate Pair Library Preparation kit (Illumina), respectively. Mate-pair libraries with larger insert sizes were constructed using a Nextera Mate Pair Library Preparation kit (Illumina). Ten micrograms of genomic DNA were tagmented in a 400 μl reaction and fractionated using SageELF, with the recovery of 11 fractions with 3–16+ kb. Each fraction was circularized and fragmented with a Covaris S2. Biotin-containing fragments were purified using Dynabeads M-280 streptavidin beads. Sequencing adapters (KAPA TruSeq Adapter kit) were attached using a KAPA Hyper Prep kit. The libraries were amplified for 10–13 cycles and purified with 0.8× AMpure XP. DNA libraries were then sequenced (~388× coverage) using Illumina HiSeq2000 (TruSeq libraries) and HiSeq2500 (Nextera libraries) at the Functional Genomics Center Zurich (FGCZ), University of Zurich, Switzerland (Supplementary Table 1).

### Genome assembly

Adapters and low-quality bases for all paired-end and mate-pair reads were removed using Trimmomatic^91^. The filtered paired-end reads of the 170 bp library were used to identify the genome size using k-mer distribution generated by Jellyfish^92^ that was implemented in the scripts by Joseph Ryan^42^. The raw R1 reads from paired-end 170 and 800 bp libraries (clipped at 95 bp, representing about 70 genome equivalents) were used to estimate the heterozygosity using KAT^43^ with a k-mer size of 23 nt. De novo genome assembly of all reads was performed using ALLPATHSLG assembler v52488^40^.

### Assembly verification and assessment of the assembled genome

#### Assembly validation

To validate the genome assembly, we mapped (i) the short reads used for the genome assembly, (ii) scanned the assembly for the presence of single-copy orthologs, and (iii) mapped transcriptome sequences obtained from seven organs.

#### Assembly verification by mapping of short reads

For each library used for genome assembly, all trimmed reads were aligned to the assembled *S. leprosula* genome using Burrows–Wheeler Aligner (BWA) v0.7.12^93^. Then, mapping ratio was calculated for each BAM file using Samtools^94^ with “flagstat” command.

#### Identification of highly conserved single-copy orthologs

BUSCO v3.1.0^42^ was run with the Embryophyta dataset and *Arabidopsis* as the species for AUGUSTUS prediction (see subsection below “Protein-coding gene prediction”).

#### Assembly verification by mapping transcriptome sequences

For mapping transcriptome sequences, samples of seven organs (leaf bud, flower bud, flower, inner bark, small seed, large seed, and calyx) were obtained from the *S. leprosula* individual used for the genome sequencing (Supplementary Table 2). Total RNA was extracted from each sample using RNeasy Plant Mini Kit (Qiagen) and it was treated with Turbo DNase I (Takara). Library preparation was carried out using a TruSeq RNA Library Preparation kit (Illumina). Paired-end sequencing was conducted for all the libraries using Illumina HiSeq2000 at the FGCZ, University of Zurich, Switzerland. Adapters and low-quality bases for all paired-end reads were removed using Trimmomatic. The trimmed sequences of each library were mapped onto the assembled genome using STAR aligner v2.4.2a^95^, and mapping ratio was obtained from the output file of STAR.

### Genome annotation

#### Repeat sequence analysis

Both homology-based and de novo prediction analyses were used to identify the repeat content in the *S. leprosula* assembly. For the homology-based analysis, we used Repbase (version 20120418) to perform a TE search with RepeatMasker (4.0.5) and the WuBlast search engine. For the de novo prediction analysis, we used RepeatModeler to construct a TE library. Elements within the library were then classified by homology to Repbase sequences (see subsection below “Preparation of repeat sequences for evidence-based gene prediction”).

#### Protein-coding gene prediction

*S. leprosula* protein-coding genes were predicted by AUGUSTUS v3.2^45^. For ab initio gene prediction, we used a pre-trained *A.* *thaliana* metaparameter implemented in AUGUSTUS. For the evidence-based gene prediction, we used the information of exon, intron and repeat sequences of *S. leprosula* as hints for the AUGUSTUS gene prediction. The details of the preparation of the hints were described in the following subsections.

#### Preparation of repeat sequences for evidence-based gene prediction

We used RepeatModeler to construct a de novo library of repeated sequences in the *S. leprosula* assembly. Then, using RepeatMasker, we generated a file containing the information of the positions of repeat sequences in the *S. leprosula* genome based on the RepeatModeler library. Elements within the library were then classified by homology to Repbase sequences. Finally, the hint file for repeat sequences in GFF format was prepared using the two scripts, “10_makeGffRm.pl” and “12_makeTeHints.pl”, stored in https://gitlab.com/rbrisk/ahalassembly.

#### Preparation of the exon and intron information for evidence-based gene prediction

To obtain the exon and intron hints, we used the mapping data of RNA-seq obtained from seven organs of the sequenced *S. leprosula* individual as described above. First, we merged all the mapping data stored in different BAM files into a single BAM file using SAMtools. Then, we prepared the intron hint file in GFF format using the, “bam2hints” script of AUGUSTUS. The exon hint file was also generated from the merged BAM file using the two AUGUSTUS scripts, “bam2wig” and “wig2hints.pl”. To conduct evidence-based gene prediction with AUGUSTUS, the three hint files (repeat sequences, intron and exon) described above were merged into a single file in GFF format.

#### BUSCO analysis

Genome annotation completeness were assessed with BUSCO v3.1.0^44^ using the Embryophyta odb9 dataset composed of 1440 universal Embryophyta single-copy genes. We referred to these 1440 genes as core genes in the main text.

#### Comparison with the proteome of *Theobroma cacao*

*T. cacao*’s gene models^18^ were downloaded from Phytozome 11 (https://phytozome.jgi.doe.gov/pz/portal.html). Then, comparison was conducted with BLASTP^96^ using the *T. cacao* proteomes as the BLAST database (E-value cutoff: 1.0E-10). Only the best hit was stored for each gene. We considered these best hits of the *T. cacao* genes as orthologs of the *S. leprosula* genes. When the *T. cacao* orthologs were identified by the BLASTP search, the orthologs of *A.* *thaliana* were defined based on the *T. cacao*-*A. thaliana* orthologous information provided by Phytozome 11 (Supplementary Table 4**)**. When the *T. cacao* orthologs were not identified, the orthologs of *A. thaliana* were searched by BLASTP (E-value cutoff: 1.0E-10) using the *A. thaliana* proteomes obtained from TAIR 10 (https://www.arabidopsis.org) as the BLAST database.

#### Synteny analysis

Based on the result of the above BLASTP searches, we assessed synteny between the *S. leprosula* scaffolds and the *T. cacao* chromosomes using MCScanX^97^. Genome information of *T. cacao* in GFF format was also obtained from Phytozome 11 as described above, which was used as an input file for MCScanX.

### Assessment of the genome assembly

#### Population data and other dipterocarp species

To assess whether the genome assembly could be used as a reference for the *S. leprosula* individuals from various populations, we checked mapping ratio, SNP positions, and admixture using the distribution-wide *S. leprosula* samples. Similarly, to assess whether the *S. leprosula* assembly could be used as a reference for aligning data from closely related species and determining their mapping ratios. For interspecific analysis, the following three Dipterocarpoideae species: *S. platycarpa*, *D.* *aromatica*, and *N.* *heimii* were used (Supplementary Table 7).

#### Sample collection and DNA extraction

Leaf samples of 19 *S. leprosula* individuals from different populations and three other dipterocarp species (*S. platycarpa*, *D.* *aromatica*, and *N.* *heimii*) were used as described in Supplementary Tables 6 and 7. Genomic DNA was extracted using the same method as described above.

#### Library preparation and sequencing

Paired-end genomic libraries (200 bp) were prepared using a TruSeq DNA Library Preparation kit (Illumina). DNA libraries were then sequenced (~16× coverage each) using Illumina HiSeq2000.

#### Mapping and SNP calling

Adapters and low-quality bases from resequencing reads were removed using Trimmomatic. All trimmed reads were then mapped and aligned to the *S. leprosula* assembly using BWA. Variants were called using GATK v3.5^98^. Duplicated reads were marked using Picard 2.6.0. Within GATK, HaplotypeCaller was used to identify variants for each sample by generating an intermediate genomic variant call format (gVCF). Subsequently, gVCF files were merged using GenotypeGVCFs to produce a raw VCF file containing SNPs and INDELs. Low-quality variants were removed from the raw VCF file by applying the hard filters implemented in GATK. Variants with genotype quality (GQ) < 20 were discarded, to capture confident genotypes with 99% accuracy. INDELs were discarded and only biallelic SNPs were retained for subsequent analysis.

#### Conservation of the predicted genes in population samples and other dipterocarp species

To check whether the predicted genes are conserved, we used the variant data obtained by resequencing the population samples and three dipterocarp species described above. After variant calling and quality filtering, Beagle v4.1^99^ was used for genotype phasing and imputing missing genotypes. Using in-house scripts, we aligned all genes from the phased data with reference to our predicted genes (.gff3 format). After the alignment, if a gene in a sample had less than 30% of ambiguous regions (missing data or less than 5× coverage), we considered that the gene existed in the sample. Then, if the gene was present in all the sequenced samples, it was considered as conserved.

#### Estimation of nucleotide diversity, Watterson’s theta and Tajima’s *D* for the predicted genes

To quantify genome-wide polymorphisms of *S. leprosula*, two measures were calculated: π, nucleotide diversity, i.e., the average number of pairwise nucleotide differences per site between sequences in a sample^100^; and *θ*_w_, intraspecific diversity, which is based on the number of polymorphic sites in a sample of sequences but is independent of their frequency^101^. The analyses were implemented using the *Compute* program from the *libsequence* package^102^. We also calculated Tajima’s *D* (*D*), an index of frequency spectrum^101^.

#### Admixture analysis

For genetic admixture analysis, we used the raw VCF file obtained from GATK as described above. VCFtools^103^ was used for additional variant filtration. First, we retained variants that were successfully genotyped in 50% of individuals and had a minimum quality score of 30, a minor allele count of 3, and a minimum depth for a genotype call of 3. Subsequently, we restricted the set to variants that were called in a high percentage of individuals (95%), a set mean depth of genotypes of 20, and a minor allele frequency of 0.05. Only biallelic SNPs were retained for subsequent analysis. PLINK v1.9^104^ was used to convert the filtered VCF format into the PLINK format (.bed/.bim/.fam) as input for ADMIXTURE v1.3^105^.

### Assessment of whole-genome duplication (WGD)

#### Dotplot analysis

Collinearity dotplot between *T. cacao* chromosomes and *S. leprosula* scaffolds (Fig. 2a) were generated by VGCS v2.0^106^. To visualize two sets (set 1 and 2) of the collinear blocks along the *T. cacao* chromosomes, we changed the order of the *S. leprosula* scaffolds and their orientation based on the results of MCScanX under the assumption that there is complete collinearity between the two species and that each *S. leprosula* scaffold was used only once for the analysis (Supplementary Table 9).

#### Ks analysis between duplicated genes and between orthologs

To conduct Ks analysis, we first identified duplicated genes and orthologs. Based on the collinear blocks and collinear genes obtained by MCScanX, groups of genes showing a 1:1 or 1:2 relationship between *T. cacao* and *S. leprosula* were identified as orthologs. In this study, the two *S. leprosula* genes within each 1:2 orthologous group were identified as duplicated genes (paralogs) created by the WGD (Supplementary Tables 4 and 10), which we referred to as “WGD-retained duplicates”. In contrast, the *S. leprosula* genes showing a 1:1 orthologous relationship was defined as “Non-retained genes”. To understand the timing of duplications, we estimated Ks between the duplicates using the *S. leprosula* genome data and transcriptome data from 10 other dipterocarp species. Furthermore, to understand the timing of the divergence of the species, we estimated the Ks between orthologs using the *T. cacao* and data from the other dipterocarp species. The details are described in the following subsections.

#### Sample collection, RNA extraction, and sequencing for the 10 dipterocarp species

We collected calyxes of fruits of the following 10 dipterocarp species in FRIM: *Dipterocarpus costulatus*, *D.* *aromatica*, *Dryobalanops oblongifolia*, *Hopea wightiana*, *N.* *heimii*, *Shorea kunstleri*, *Shorea sumatrana*, *Upuna borneensis*, *Vatica odorata*, and *Vatica umbonata* (Supplementary Table 11**)**. The calyx samples were immersed in RNAlater (Ambion) immediately after harvesting and stored at −20 °C. RNA was extracted using the CTAB method^90^. DNA was removed with Turbo DNase I (Takara). Purification was conducted using the RNeasy Plant Mini Kit (Qiagen). Paired-end sequencing was conducted for all the libraries using Illumina HiSeq2000.

#### Transcriptome assembly for the 10 dipterocarp species

Before the assembly of the transcriptome, sequences with low-quality bases were removed using Trimmomatic with a parameter set to “HEADCROP:10 LEADING:3 TRAILING:3 SLIDINGWINDOW:4:15 MINLEN:36”. Using the trimmed sequences, de novo transcriptome assembly by Trinity assembler (version r20140413p1)^107^ was conducted for each species with a default parameter. The numbers of reads before and after trimming, and those of the obtained contigs by assembly are found in Supplementary Table 11.

#### Identification of orthologs for the 10 dipterocarp species

Protein sequences of the genes for the 10 dipterocarp species were obtained with TransDecoder. The reciprocal BLASTP best hits (E-value cutoff: 1.0E-10) between the predicted *S. leprosula* and each dipterocarp species’ proteins were identified as orthologs.

#### Estimation of Ks

The Ks between each homologous (orthologous or paralogous) gene pair was estimated as follows. For each gene pair, first, the amino acid sequences were aligned using BLASTP. Then, the alignments were edited by retaining the aligned positions only if the three aligned positions both upstream and downstream did not contain any alignment gaps. Alignments were also retained only if they were longer than 150 aa and covered at least half of the length of both amino acid sequences. When estimating the Ks between orthologous gene pairs in orthologous groups with a 1:2 relationship, the orthologous gene pair producing the longer alignment was used. Nucleotide alignments of the coding sequences were created using the amino acid alignment as a guide, and the Ks was estimated using the coding sequences by CODEML with the Yang and Nielsen model from the PAML package^108^ with the following parameters: model = 0, NSsites = 0, fix_alpha = 1, alpha = 0, fix_kappa = 0, RateAncestor = 0, CodonFreq = 2. For each of the 10 other Dipterocarpaceae species, Ks was estimated between the hits identified by all against all BLASTP according to the criteria outlined above. When the pairwise Ks are estimated between all paralogs, if a particular gene is duplicated multiple times, the Ks of the same duplication events will be estimated multiple times. As such, when obtaining Ks distributions for the 10 other Dipterocarpaceae species, single Ks estimates representing each duplication event were obtained by clustering the paralogs into gene families based on the Ks estimates as previously described^48^.

### Time estimation of the WGD event

#### Preparation of a gene set for phylogenetic dating of WGD

Based on the orthologs and paralogs identified above, 204 orthologous gene families were created for each *S. leprosula* WGD duplicate pair. Starting with *S. leprosula* WGD duplicate pairs with Ks = 0.2–0.6, a *T. cacao* ortholog was added if the Ks between the *T. cacao* and *S. leprosula* orthologs was 0.5–1.2. For both *S. leprosula* genes, one ortholog from either *D. aromatica* or *D. oblongifolia*, and one ortholog from *U. borneensis*, *V. odorata*, or *V. umbonata* were added if the Ks between the orthologs was 0.05–0.30. If multiple orthologs were present, the gene with the lowest amino acid divergence (Ka, estimated together with the Ks as described above with its *S. leprosula* ortholog was chosen. Finally, *Gossypium raimondii* genes identified as collinear orthologs with *T. cacao* by the PLAZA database^109^ were added only if the *T. cacao* gene corresponded to one or two *G. raimondii* genes. If there were two *G. raimondii* genes, the gene with a lower amino acid divergence with the *T. cacao* ortholog based on the amino acid alignment was chosen, as the alignments are more likely to be reliable. Thus, all orthologous gene families contained two *S. leprosula* genes, one *T. cacao* gene, one *G. raimondii* gene, two duplicates of either *D. aromatica* or *D. oblongifolia*, and two duplicates of *U. borneensis*, *V. odorata*, or *V. umbonata* (see Fig. 3a). For each orthologous gene family, the amino acid sequences of each gene were aligned using MAFFT version 7^110^ with the alignment option *linsi*. The alignments were cleaned by removing poorly aligned positions and divergent regions using Gblocks version 0.9b^111^, and gene families with a remaining alignment length of at least 100 aa were retained for further phylogenetic dating.

#### Phylogenetic dating of WGD

Phylogenetic dating was performed on each orthologous gene family using the BEAST package v1.8^112^ following the method previously described^47^. Briefly, an uncorrelated relaxed clock model that assumes an underlying log-normal distribution (UCLD) was used, whereas the Le-Gascuel (LG) substitution model^113^ with gamma-distributed rate heterogeneity across sites using four rate categories^114^ was set as the underlying evolutionary model. A Yule pure birth process^115^ was specified for the underlying tree model, and a uniform prior between 0 and 100 for the Yule birth rate was used. An exponential prior with mean 0.5 on the rate heterogeneity parameter, mean 1/3 on the standard deviation of the UCLD clock model, and a diffuse gamma prior with shape 0.001 and scale 1000 on the mean of the UCLD clock model were used. The BEAST files (.xml) that were used to run without data under the two different calibration settings (see below) are provided as Supplementary Data 3. The MCMC analysis for each orthologous gene family was run for 10 million generations while sampling every 1000 generations, resulting in a total of 10,000 samples per family. The topology was fixed according to the widely accepted phylogenetic relationship shown in Fig. 3a. The calibrations and constraints are described in detail below.

The resulting files of each family were processed with LogAnalyser, which is part of the BEAST package, with a burn-in of 1000 samples, and only those with a minimum effective sample size (ESS) of at least 200 for all statistics were retained. For the files retained, the median ages were used to represent the age of each node. Although one family in Setting 2 (FamilyID 85 in Supplementary Table 12) was removed as it had very low (<200) ESS for multiple statistics, all the remaining families had an ESS of more than 200 for all statistics. Subsequently, for nodes 1–5, age distributions of the median age estimates of each family were obtained. Then, the kernel density estimate (KDE) of the ages of all the families was calculated using the R density function, and the mode was used as the consensus age of each node. Finally, in order to obtain 95% CIs of the consensus age of each node, 1000 bootstrap datasets of the age estimates of each family were created, and the mode of the KDE was calculated for each bootstrap dataset, as described in a previous study^47^. Then, the modes of the 26th and 974th bootstrap density estimate (ranked in order of increasing value of their mode) were taken as the lower and higher 95% CI boundary, respectively.

#### Calibrations and constraints for phylogenetic dating of WGD

The nodes corresponding to the divergence of *T. cacao* and *G. raimondii* (node 2) and the divergence of *Shorea* and *Dryobalanops* (node 5) were both constrained based on fossil records. A minimum age of 55.8 Ma was assigned to the *T. cacao*–*G. raimondii* node (node 2) based on the fossil from the middle-to-late Paleocene that has been attributed to the Eumalvoideae^22,116^. A minimum age of 34 Ma was assigned to the *Shorea*–*Dryobalanops* node based on fossils from the late Eocene attributed to *Shorea*^74^, which, to our knowledge, were the earliest fossils that could be confidently attributed to *Shorea*. Log-normal prior distributions with the means equal to the minimum fossil age plus 10% were assigned to the *T. cacao-G. raimondii* and *Shorea-Dryobalanops* nodes. These correspond to 61.38 Ma for *T. cacao*–*G. raimondii* (mean = 1.719, offset = 55.8) and 37.4 Ma for *Shorea*–*Dryobalanops* (mean = 1.22, offset = 34), with a standard deviation of 1 for these two nodes^63^. Although there were no appropriate fossil calibrations that could be assigned to the root node, the divergence between Dipterocarpaceae and Malvaceae has been estimated as ∼70–80 Ma by previous phylogenetic dating studies^22,64^. To incorporate this information, a normal prior distribution with a mean of 75 Ma and a standard deviation of 8 was assigned to this node as a secondary calibration.

Considering the various uncertainties associated with the fossil and secondary calibrations, an alternative set of calibrations (Setting 2) was used to perform phylogenetic dating. In particular, we considered that the settings described above (Setting 1) may be slightly biased toward producing younger age estimates; therefore, we applied a setting that allows each node to explore age estimates that are older. For instance, the true divergence date can potentially be a lot older than the fossil records used as lower bounds. In addition, a fossil from, e.g., the late Eocene can be anywhere between ∼34 and ∼41 Ma. Thus, assigning a prior distribution with a mean that is only a few million years older than the youngest possible date of the fossil can be argued as being rather restrictive. Similarly, we considered the possibility that previous estimates of the divergence dates between Dipterocarpaceae and Malvaceae are underestimated due to the limited sampling of Dipterocarpaceae and/or the low substitution rates of woody lineages such as Dipterocarpaceae^117^. In fact, the posterior age distribution of the root node from Setting 1 was a lot older than the prior age distribution. As such, a log-normal prior distribution with the mean age corresponding to 70.7 Ma and a standard deviation of 0.25 (offset = 55.8, mean = 2.7), based on the phylogenetic dating results of Malvaceae^118^, was assigned to the *T. cacao*–*G. raimondii* node, a log-normal prior distribution with the mean age corresponding to 42 Ma and a standard deviation of 0.5 (offset = 34, mean = 2.08) was assigned to the *Shorea*–*Dryobalanops* node, and a log-normal distribution with the mean age corresponding to 78.2 Ma and a standard deviation of 0.3 (offset = 55.8, mean = 3.11) was assigned to the root node. The marginal prior densities for each node based on running the MCMC sampler without data are shown in Supplementary Fig. 9 for both parameter settings.

One recent study performed the most comprehensive molecular dating of Dipterocarpaceae to date^23^. These authors chose not to use any fossil constraints citing difficulties to assign dipterocarp fossils to particular clades, and argued that the fossil ascribed to *Shorea*^74^ that we used as a fossil constraint is likely to be of another a species within *Anthoshorea*. This does not affect our result as *Shorea* and *Anthoshorea* share a more recent common ancestor the node 5 that we applied the fossil constraint to. These authors instead used a log-normal distribution with a mean of 87.5 Ma as a calibration point to the most recent common ancestor of Dipterocarpoideae and Sarcolaenaceae, which is more recent than the root node in our study. This is based on a widely cited assumption among dipterocarp researchers that Sarcolaenaceae, which is endemic to Madagascar, diverged from its sister species due to the separation of India and Madagascar ~87.6 Ma^119^. We chose not to incorporate this age as prior information considering the results of many other plant groups suggesting an important role of dispersal over vicariance in explaining trans-oceanic distributions (see “Discussion”). We note nevertheless that this age is compatible with our results if we assume that this divergence occurred shortly after the divergence of Dipterocarpaceae and Malvaceae.

We also note that some studies have assumed that *Vateriopsis*, which is endemic to the Seychelles, diverged from its sister lineage containing *Vatica*, *Upuna*, and *Vateria* ~63 Ma by the separation of the Seychelles and India^63^, leading to much earlier estimates for the divergence between the lineages of Dipterocarpoideae (e.g., ∼80 Ma for node 4 and ∼55 Ma for node 5 or ∼95 Ma for node 4 and ∼70 Ma for node 5)^63,120^, compared with our estimates of Fig. 3a (~42–50 Ma for node 4 and ~36–40 Ma for node 5). By contrast, the aforementioned comprehensive molecular dating study of Dipterocarpacae reported mean age estimates of 54.9 Ma for node 4 and 43.3 Ma for node 5, but both with posterior density intervals of ~30 Ma^23^. These estimates are similar to our estimates, and these authors suggested that the divergence of *Vateriopsis* occurred most likely by long-distance dispersal rather than vicariance.

### Characterization of duplicated genes

#### Ka/Ks analysis

Previous studies suggested that ancient homologs tend to show slower evolutionary rates at nonsynonymous sites^49,50^. We assessed whether the Ka, Ks, and Ka/Ks of the WGD-retained duplicates were significantly smaller than those of the non-retained genes between *S. leprosula* and *T. cacao* (Malvaceae), *S. leprosula* and *H.* *wightiana* (Dipterocarpaceae), and *S. leprosula* and *U.* *borneensis* (Dipterocarpaceae) using the same approach with CODEML in the PAML package explained above. The distributions of Ka, Ks, and Ka/Ks estimates between *S. leprosula* and *T. cacao* were compared between the WGD-retained duplicates and the non-retained genes. We also compared the Ka, Ks, and Ka/Ks of upregulated genes under no-irrigation treatment in section “No-irrigation treatment” below that are the WGD-retained duplicates (“WGD-retained drought-up genes”) with those of the non-retained genes. Statistical analyses were conducted using one-sided Mann–Whitney *U* tests considering multiple comparisons (*P*-value cutoff: 0.05 after Bonferroni correction).

#### Gene ontology (GO) enrichment test for the WGD-retained duplicates

For GO enrichment test, we used the GO information of the *A. thaliana* orthologs in Supplementary Table 4. The *A. thaliana* GO terms were downloaded from TAIR 10 (https://www.arabidopsis.org) on 21 November 2017. The GO enrichment analysis was performed using the BioConductor package topGO^121^ in R. For enrichment analysis, we adopted the “elim” algorithm together with Fisher’s statistic to test the functions of the retained duplicated genes. The “elim” algorithm scores *P*-values by considering the topology of GO graphs^122^. We listed the top 40 significant GO terms identified by the “elim” algorithm method and the *P*-values obtained (*P*-value cutoff: 0.05). To consider that different scoring methods may affect the result of significance, we also evaluated the significance of the enriched GO terms by Fisher’s exact test (“classic” algorithm in topGO) and multiple test corrections by false discovery rate (FDR cutoff: 0.05) using the Benjamini-Hochberg procedure.

#### GO enrichment test for tandemly duplicated genes

To compare with the result of GO enrichment test in the WGD-retained duplicates, we also conducted GO enrichment test for tandemly duplicated genes in the *S. leprosula* genome. For this purpose, we first identified the tandemly duplicated genes by using the following criteria: (i) neighboring genes on the same scaffold corresponded to the same gene in *T. cacao* as a result of BLASTP (see above subsection “Comparison with the proteome of *Theobroma cacao*”); (ii) one of the neighboring genes showed a 1:1 or 1:2 syntenic orthologous relationship with a *T. cacao* gene (i.e., WGD-retained duplicates or non-retained genes). In this analysis, we considered the genes that were not the tandemly duplicated genes and showed syntenic relationship with *T. cacao* genes as non-tandem duplicates and used them as a control of comparisons. The procedures of GO enrichment tests for the tandemly duplicated genes were the same as those described above.

### No-irrigation treatment

#### Experimental condition of the no water treatment

To confirm the functionality of the duplicated drought-responsive genes in the WGD-retained duplicates, an experiment was conducted on six *S. leprosula* seedlings grown in the nursery of the Forest Research Institute Malaysia (FRIM). The seedlings were about 2 years old with an average height of 36 cm and an average collar diameter of 3.55 mm. All the seedlings were transferred to a plant growth chamber (Percival PGC-15) with the following conditions—day: 29 °C, 75% humidity; night: 26 °C, 75% humidity; day/night cycle: 12/12 h. Two types of treatment were applied: 50 mL of water daily (control) and no irrigation (artificial drought). Each treatment had three replicates and lasted for 9 days.

#### Sample collection, RNA extraction, and sequencing for RNA-seq data

Leaves were sampled at day 0 (before no-irrigation treatment, at 09:00) and at day 7 (during the no-irrigation treatment, at 0900). The leaves were immersed in RNAlater (Ambion) immediately after harvesting and stored at −20 °C. RNA was extracted from the leaves using the same method described above. Library preparation was carried out using an Illumina TruSeq Stranded mRNA library preparation kit in accordance with the manufacturer’s recommendations. Paired-end 125 bp sequencing using an Illumina HiSeq2500.

#### Analysis of RNA-seq data

All data analysis was performed using the SUSHI pipeline^123^. Paired-end raw sequence reads were combined and mapped onto the *S. leprosula* genome and the annotation file using STAR aligner^87^. The mapped reads (Supplementary Table 18) were then counted using the FeatureCounts function of Rsubread^124^. A quality control step was subsequently performed on the counted reads using CountQCApp from SUSHI. We also checked for the presence of contamination or ribosomal RNA content on our reads using FastqScreenApp from SUSHI. Finally, the genes that were differentially expressed between the two time points were detected using the BioConductor package edgeR^125^ in R which based on a negative binomial distribution to model the raw read counts in a gene-wise manner and followed by Trimmed Mean of M-values (TMM) method for the sequence depth normalization^126^.

Using the output from edgeR, we split the data into two groups of upregulated and downregulated genes in response to the dry treatment. We filtered those genes based on the significance-level false discovery rate (FDR < 0.05), obtaining 1200 upregulated genes and 914 downregulated genes. Both the upregulated and the downregulated genes underwent an enrichment analysis using the BioConductor package topGO in R with the same procedures described above.

#### Comparison between drought-response and duplicated genes

To test whether the drought-response genes obtained in above are enriched in the *S. leprosula* WGD-retained duplicates, we conducted Fisher’s exact tests using the “fisher.test” function in R. Bonferroni corrections were conducted by considering multiple comparisons.

As a comparison, we also tested whether the drought-response genes are enriched in the tandemly duplicated genes in *S. leprosula*, by performing Fisher’s exact tests and Bonferroni corrections as described above.

### Distributions of *Shorea leprosula* and *Shorea roxburghii*, and the precipitation in their habitats

We downloaded the distribution data of *S. leprosula* and *S. roxburghii* (a closely related *Shorea* species to *S. leprosula* that grows in a more seasonal climate) from the Global Biodiversity Information Facility (GBIF) (https://www.gbif.org) using the “gbif” function in the R package, “dismo”. We further downloaded the precipitation data of the driest month (BIO14) from WorldClim (https://worldclim.org) at the resolution of 2.5 min by using the “getData” function in the R package, “raster” for every site of the two species in the GBIF data in the region ranging from −6° to 22° of latitude and from 90° to 120° of longitude. We combined these data to assess the distribution of the driest month for these two species growing in contrasting climates. We further analyzed 30-day cumulative rainfall for 13 years and 2 months from Danum Valley Field Centre in Sabah, Borneo (data downloaded from searrp.org) and Pasoh Forest Reserve, Peninsular Malaysia^127^ to examine the temporal rainfall and drought patterns of two sites within the distribution of *S. leprosula*.

### Statistics and reproducibility

The data of this genome study was derived from a single diploid individual *S.* *leprosula* tree B1_19 (DNA ID 214) located at the Dipterocarp Arboretum at Forest Research Institute Malaysia (FRIM). RNA-seq reads obtained from seven organs used for genome annotation were derived from the same tree. Resequencing data were derived from 19 *S.* *leprosula* individuals obtained across its distribution range in Southeast Asia (Peninsular Malaysia, Borneo, Kalimantan, and Sumatra) and three other closely related dipterocarp species (*S. platycarpa*, *D.* *aromatica*, and *N.* *heimii*). RNA-seq of 10 other dipterocarp species were obtained from Dipterocarp Arboretum at FRIM for comparative genomics and molecular dating analysis. No-irrigation treatment were conducted using 2 years old *S. leprosula* seedlings in a plant growth chamber (Percival PGC-15). Two types of treatments were applied: 50 mL of water daily (control) and no-irrigation (artificial drought). Each treatment had three replicates. All statistical tests were conducted using publicly available programs and packages as described in sections under “Methods”. Reproducibility can be accomplished by following the sample used and methods outlined above. Statistical analysis using R were described above in each section.

### Reporting summary

Further information on research design is available in the Nature Research Reporting Summary linked to this article.

## Supplementary information

Supplementary information.

Reporting summary.

## Data Availability

Raw reads and genome assembly have been deposited to DDBJ under BioProject accession numbers PRJDB8161 and PRJDB8182, respectively. All corresponding data related to this study are available at Figshare^128–132^.
