# Supplementary material for: The genome of Shorea leprosula (Dipterocarpaceae) highlights the ecological relevance of drought in aseasonal tropical rainforests
Source: Commun Biol. 2021 Oct 7;4:1166. doi: 10.1038/s42003-021-02682-1 (PMC8497594; doi:10.1038/s42003-021-02682-1)
Supplement: Supplementary file 1 — Supplementary information. [file 42003_2021_2682_MOESM1_ESM.pdf]

Ng et al. The genome of a dipterocarp *Shorea leprosula* highlights the ecological relevance of drought in aseasonal tropical rainforests. COMMSBIO-20-1594B

Supplementary figures

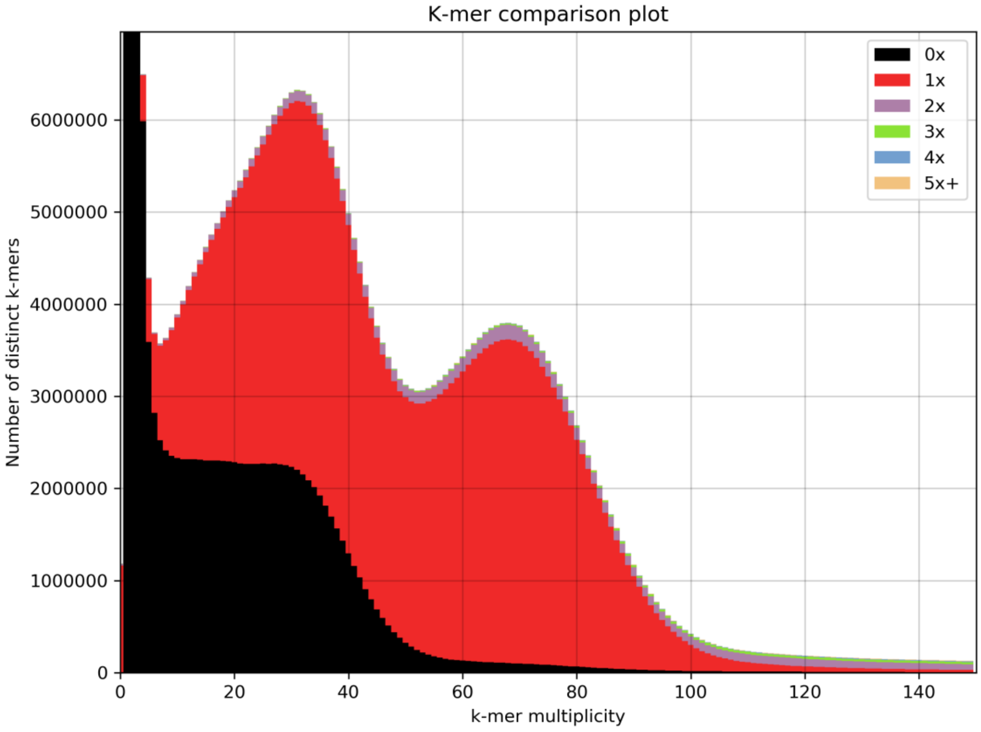

Supplementary Fig. 1: K-mer spectrum of the *Shorea leprosula* genome assembly. By scanning the raw reads, the presence of two peaks revealed a highly heterozygous genome, with the heterozygous peak being much higher than the homozygous. The frequency of the raw read k-mers in the assembly is represented by the colored areas under the curve. The assembly is haploid (i.e. the area under the homozygous peak is all red, all homozygous regions are collapsed into one in the assembly) and the heterozygote regions are represented only once (red area under the heterozygous peak), while the other variant is absent (black area under heterozygous peak).

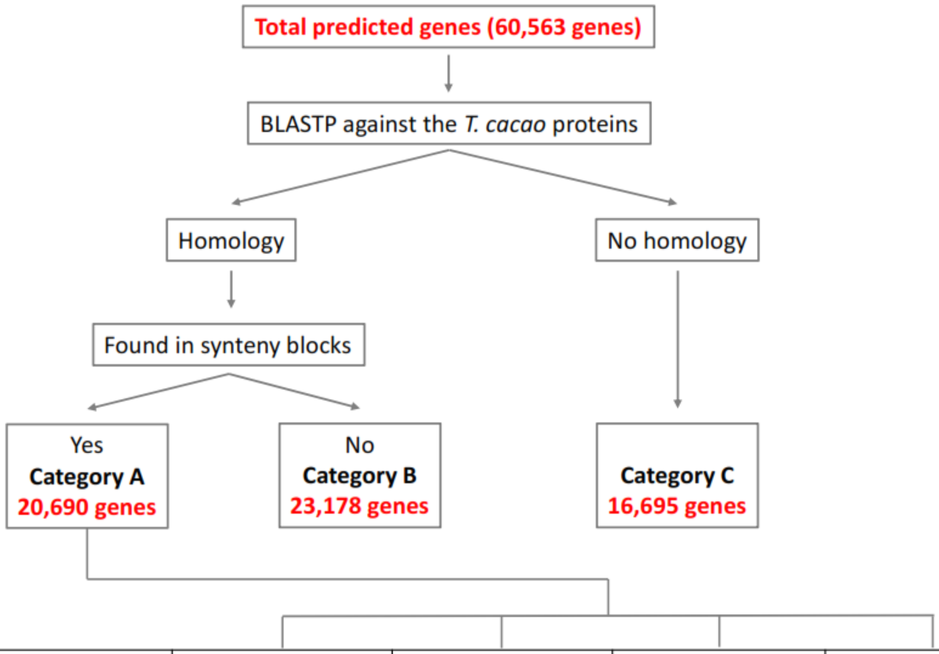

T. cacao : *S. leprosula*	1:1	1:2	1:3	1:>3
Total number of genes	7,594	12,886	198	12

Supplementary Fig. 2: The orthology of *Shorea leprosula* genes with those of *Theobroma cacao* was evaluated by sequence homology and synteny. Syntenic blocks had different ratios between *S. leprosula* and *T. cacao*.

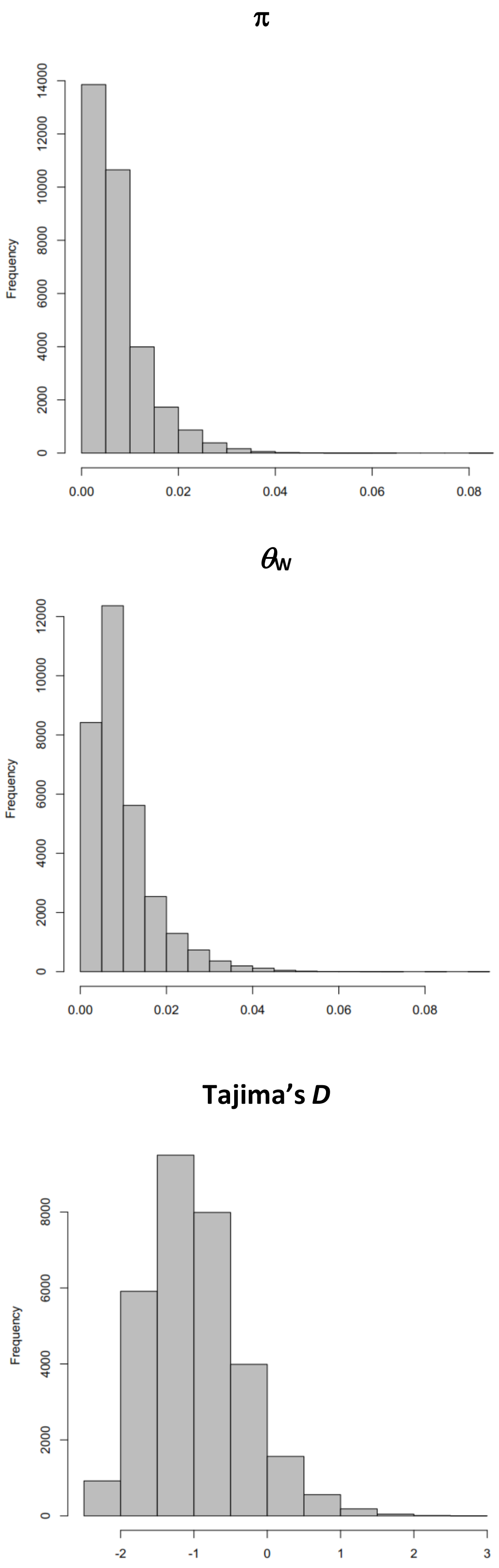

Supplementary Fig. 3: Genome-wide distribution of nucleotide diversity (π), theta Watterson (θ_W) and Tajima’s *D* values for the genes in the categories A and B.

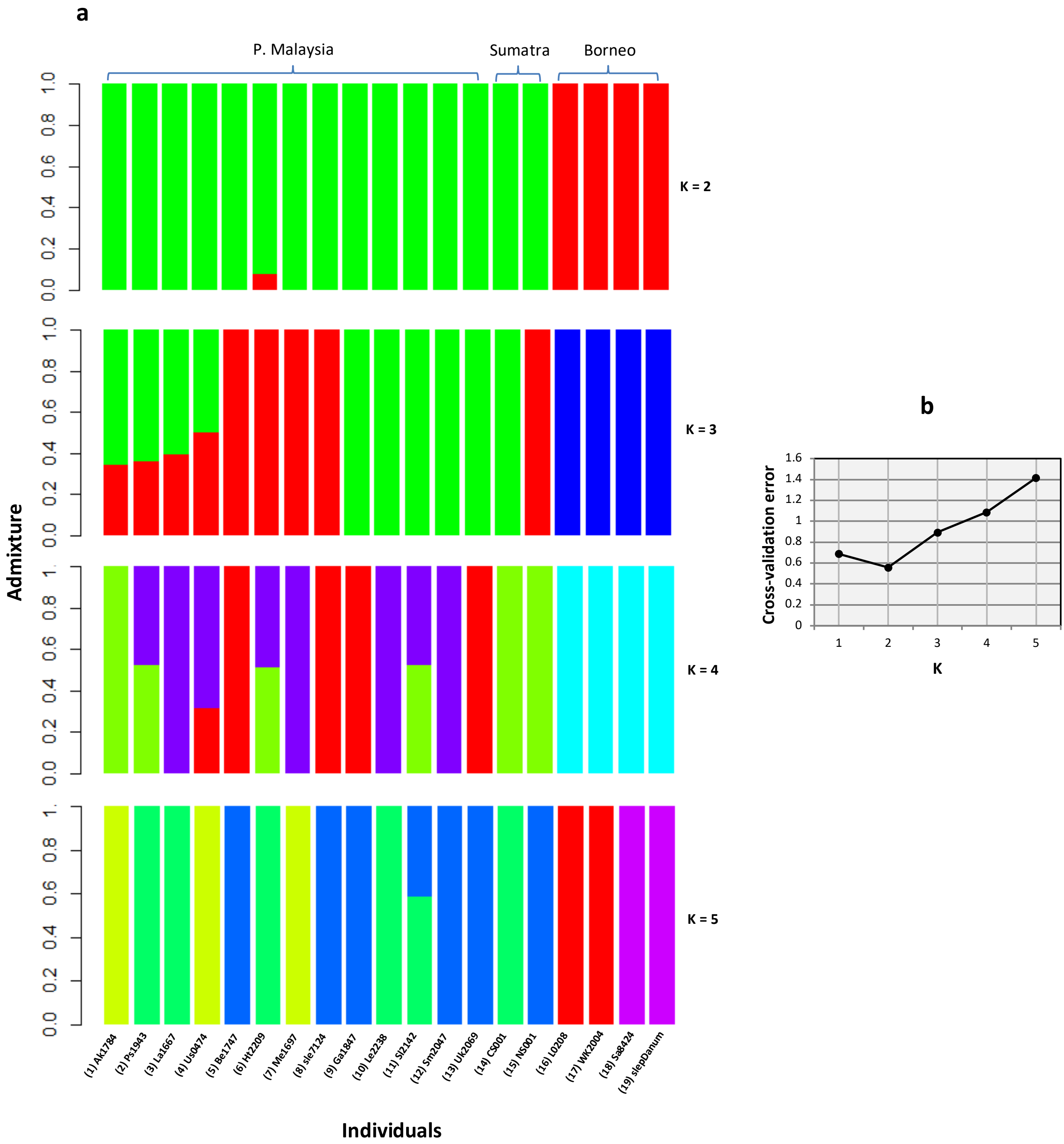

Supplementary Fig. 4: (a) Admixture analysis of 19 individuals of *S. leprosula* from P. Malaysia, Sumatra, and Borneo with K = 2–5 and (b) the cross-validation error plot suggesting K = 2 is a sensible modelling of choice.

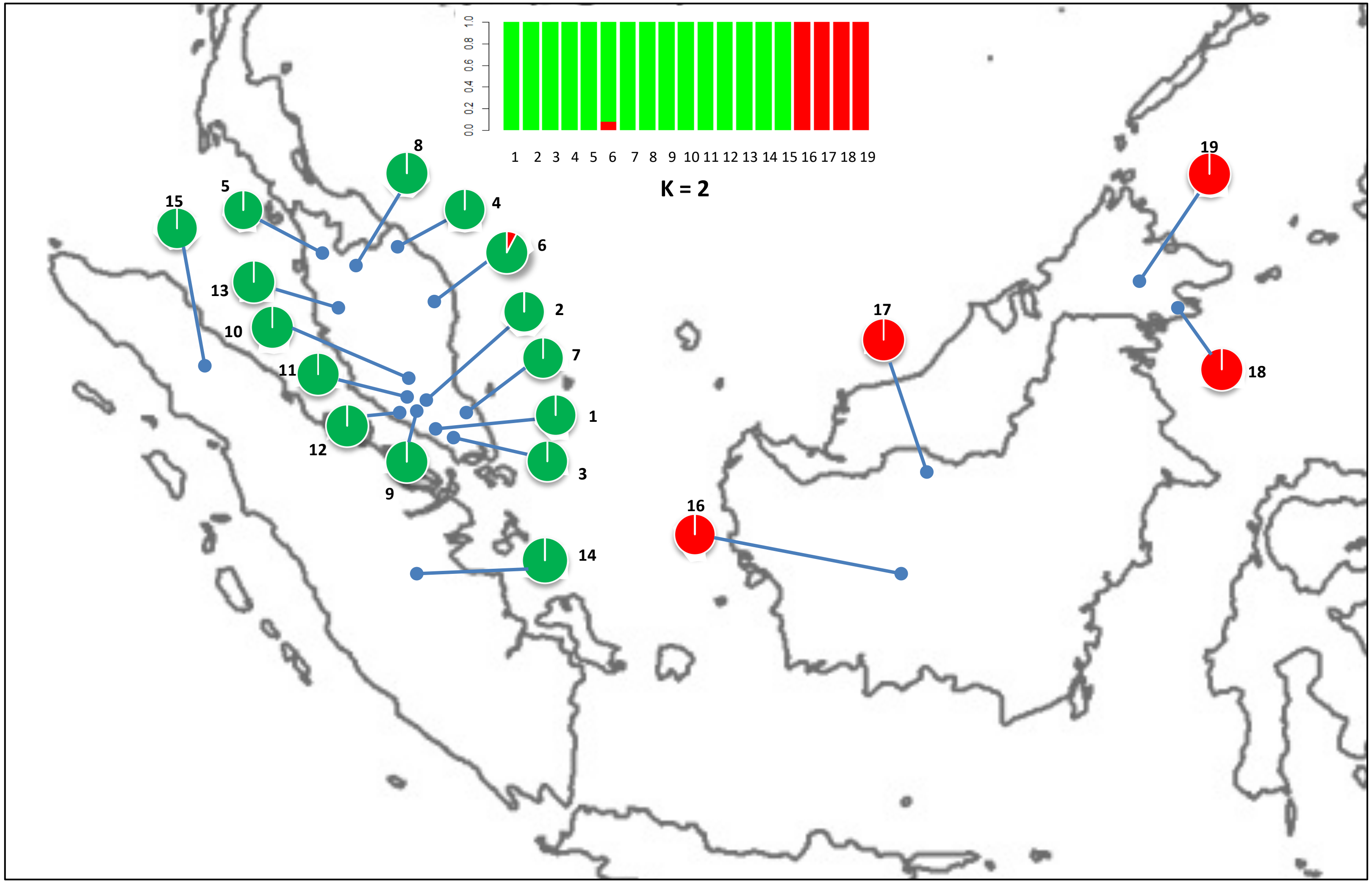

Supplementary Fig. 5: Map location of each sample and its admixture pattern based on K = 2

a

b

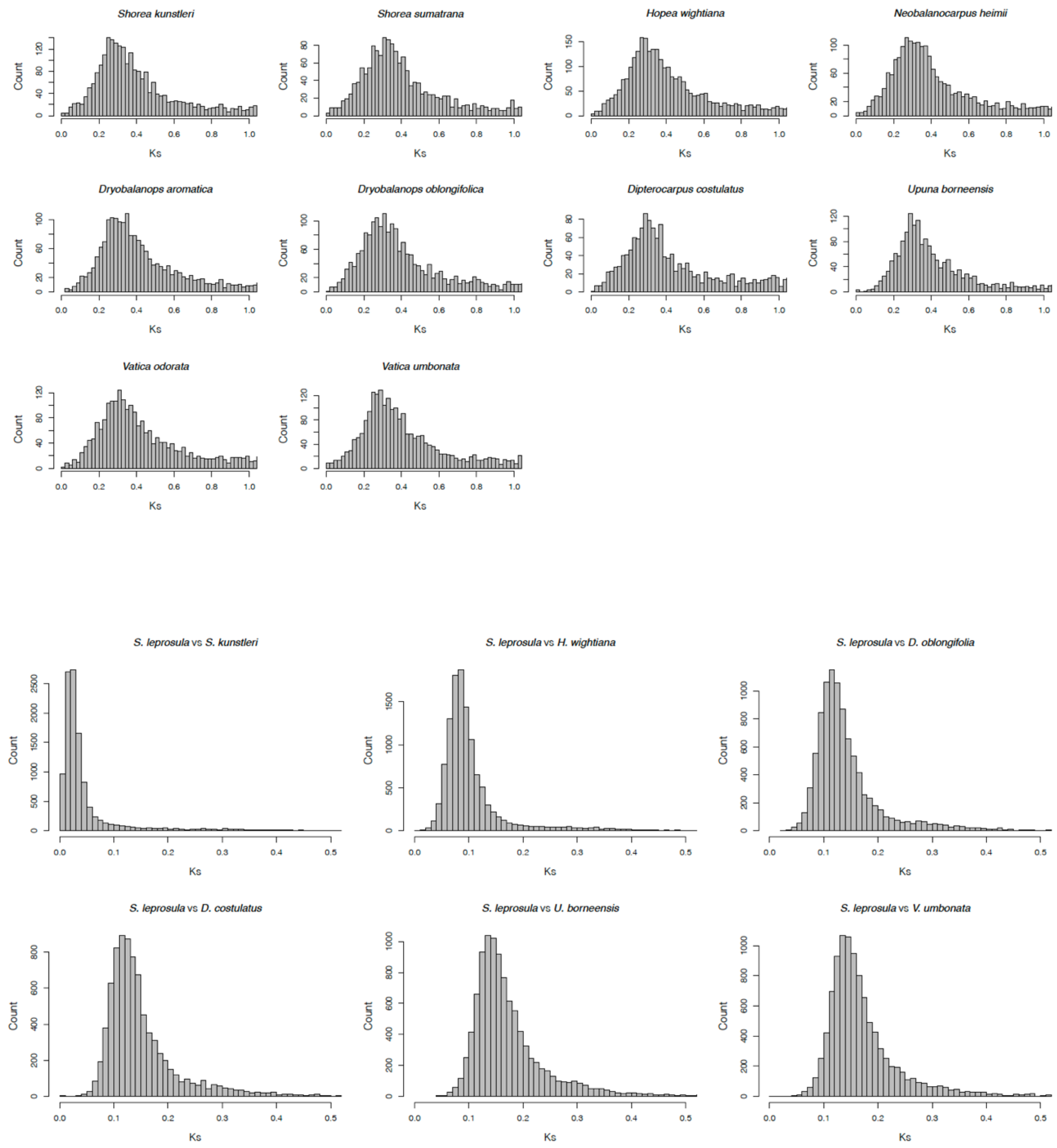

Supplementary Fig. 6: (a) Ks distribution of the duplicated genes (paralogs) identified from the transcriptome of 10 other Dipterocarpoideae species from seven different genera, and (b) Ks distribution of orthologs between *S. leprosula* and other Dipterocarpoideae species.

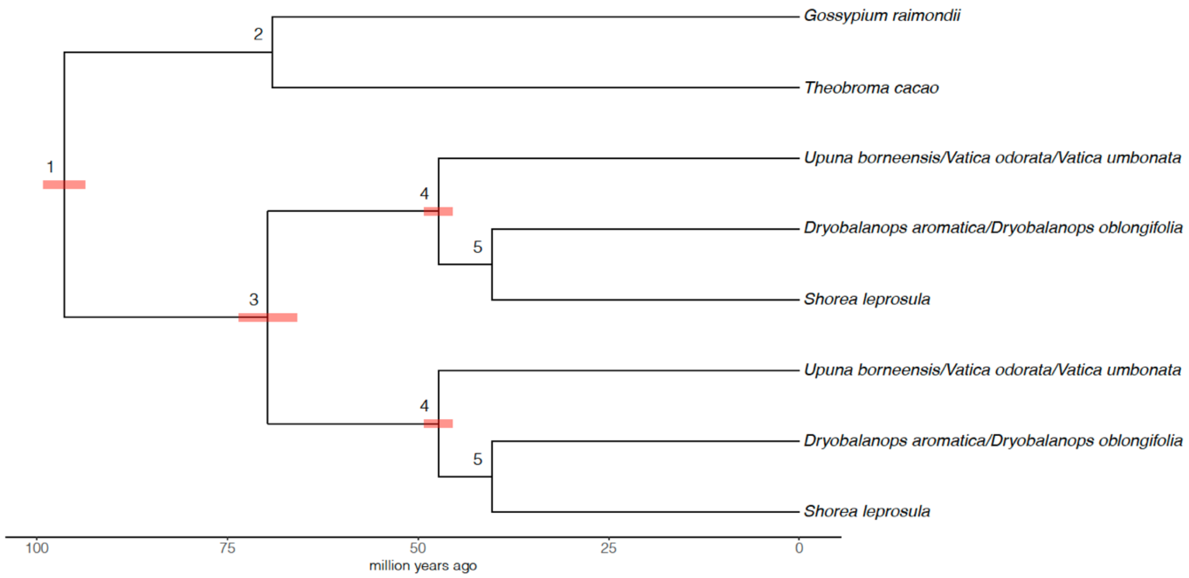

Supplementary Fig. 7: Representative gene tree used for phylogenetic dating and the estimated ages of each node based on the parameter Setting 2. The red bars correspond to the 95% confidence intervals. Only the confidence intervals with a range of > 1 million years are shown. Node 3 corresponds to the dipterocarp WGD.

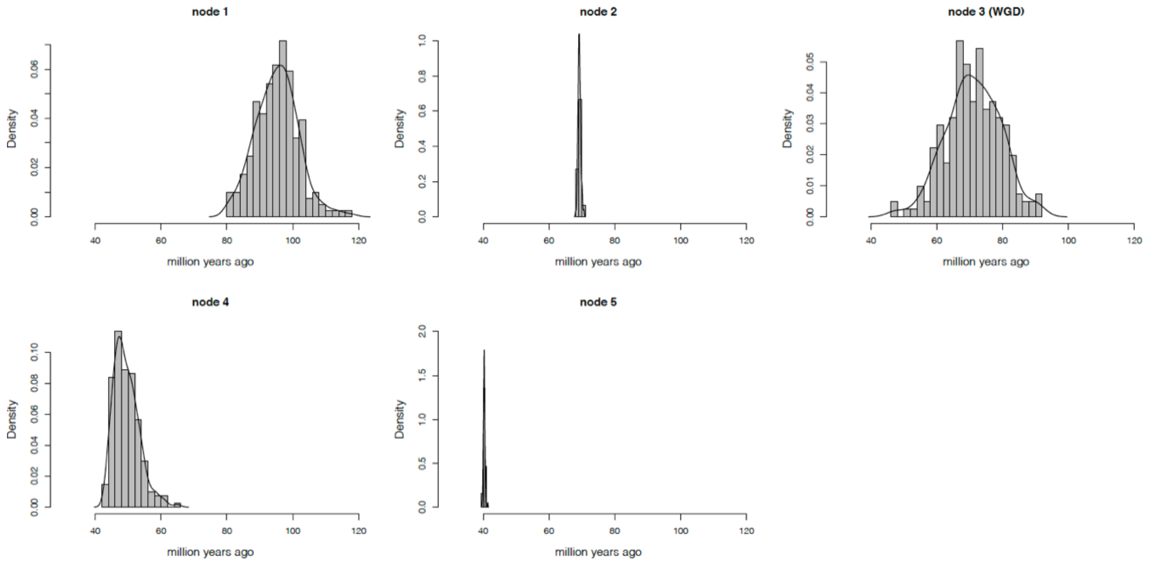

Supplementary Fig. 8: Age distribution of the divergence of each node based on the parameter Setting 2.

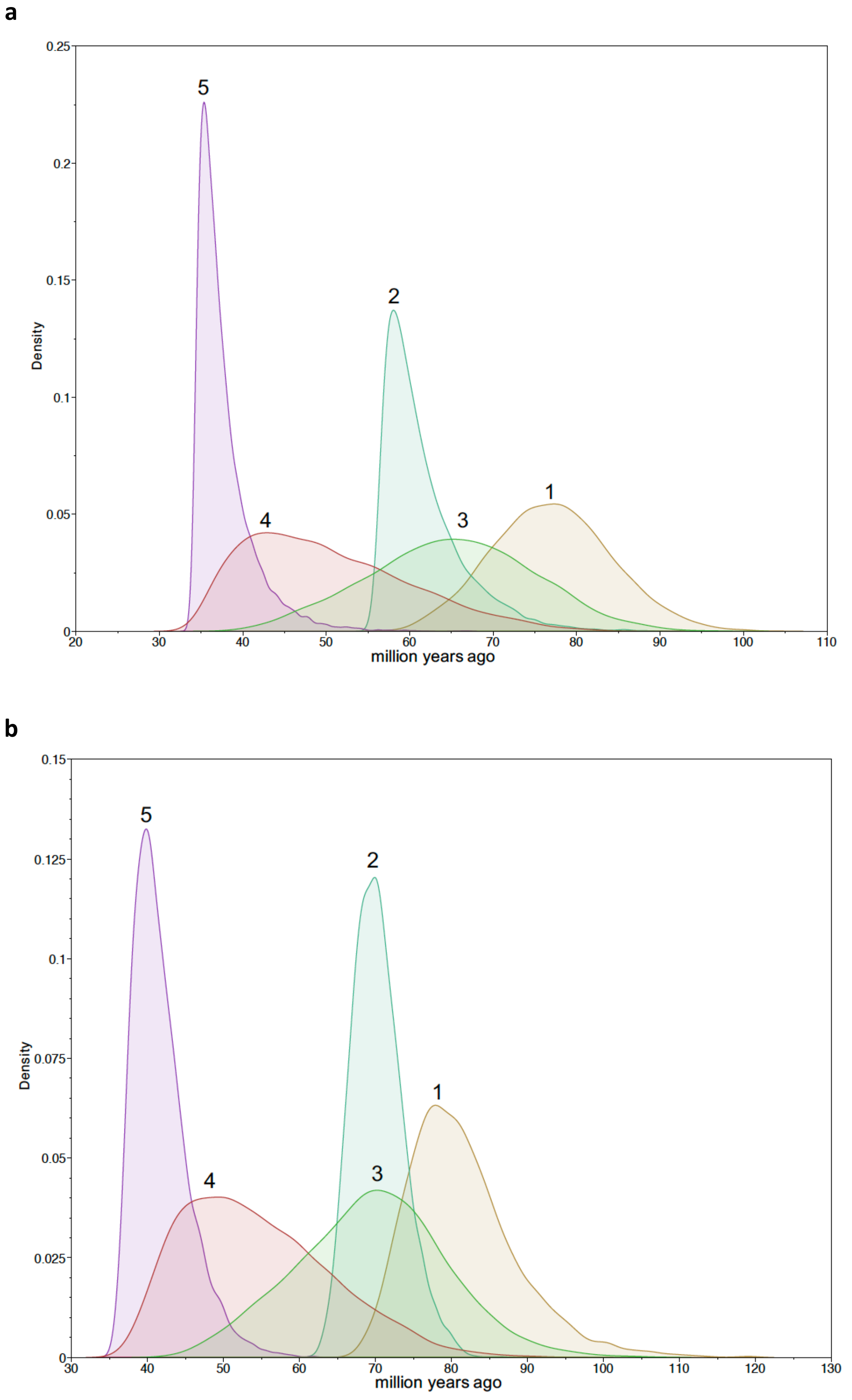

Supplementary Fig. 9: Prior densities for each node based on running the MCMC sampler without data with the parameter (a) Setting 1 and (b) Setting 2. The node numbers correspond to those in Fig. 3a.

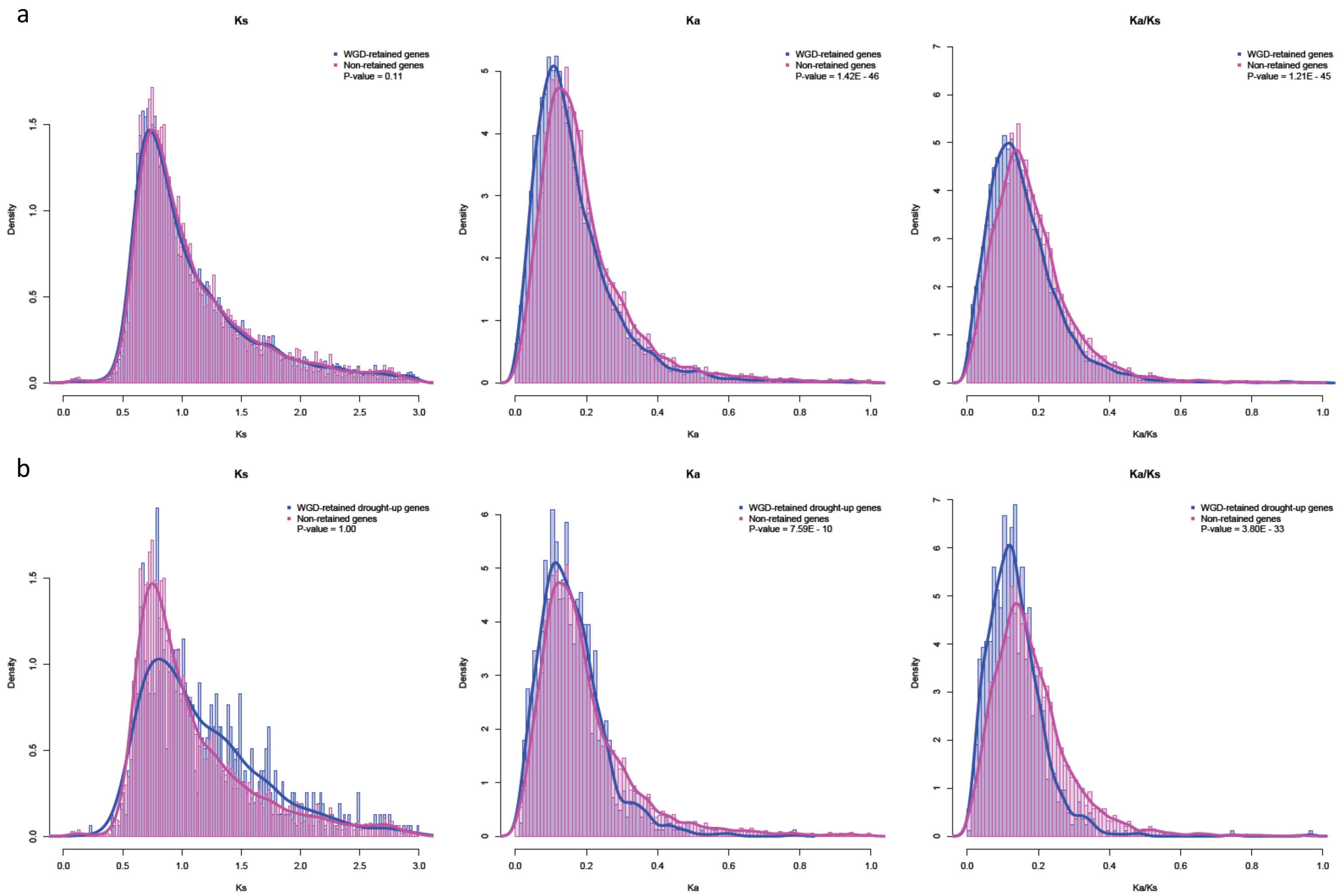

Supplementary Fig. 10: (a) Comparisons of the distributions of Ks, Ka, and Ka/Ks between the WGD-retained genes (blue) and the non-retained genes (red). (b) Comparisons of the distributions of Ks, Ka, and Ka/Ks between the WGD-retained drought-up genes (blue) and the non-retained genes (red). *P*-value: Mann–Whitney *U* test after Bonferroni correction.

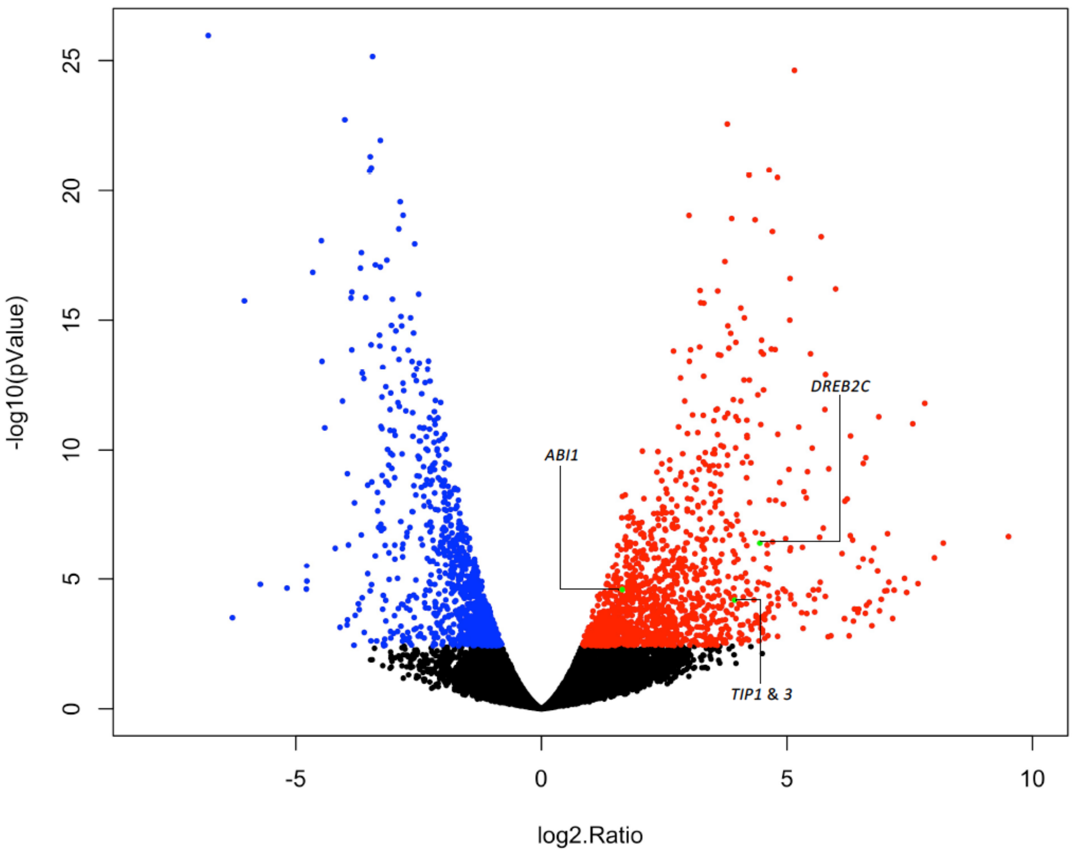

Supplementary Fig. 11: Volcano plot showing differentially expressed genes upregulated (red), downregulated (blue), genes with not significant changes (black) and duplicated genes with known drought related functions (green).

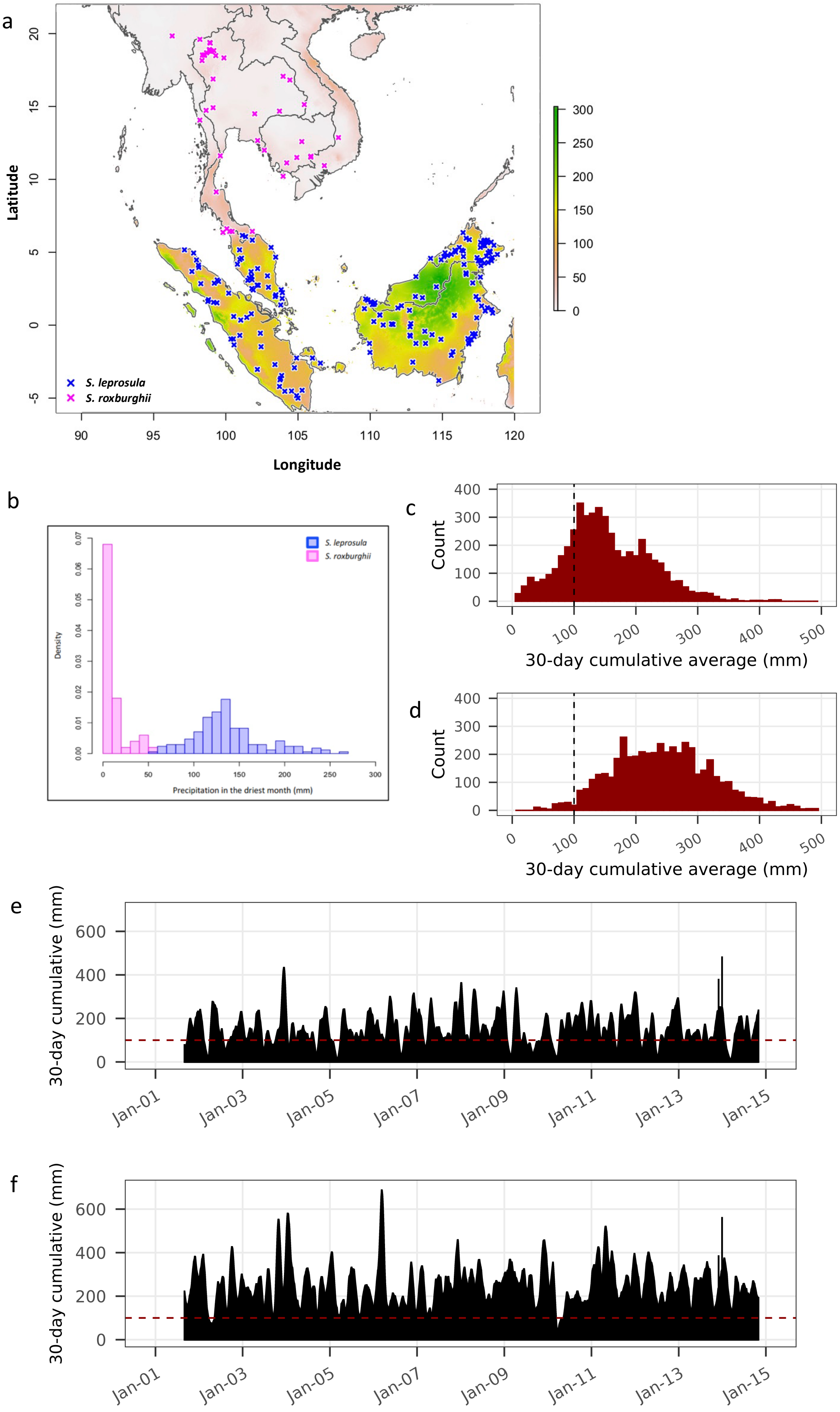
a
b
c
d
e
f
Latitude
Longitude
S. leprosula
S. roxburghii
Density
Precipitation in the driest month (mm)
Count
30-day cumulative average (mm)
30-day cumulative (mm)
Jan-01
Jan-03
Jan-05
Jan-07
Jan-09
Jan-11
Jan-13
Jan-15

Supplementary Fig. 12: (a) Distributions of *S. leprosula* (blue) in the aseasonal tropics and *S. roxburghii* (pink) in the seasonal tropics. The colors on the map indicate the precipitation of the driest months. (b) The precipitation of the driest months in the *S. leprosula* (blue) and *S. roxburghii* (pink) populations. The y-axis of the histograms was represented as probability density. (c,d) Histogram of 30-day cumulative average rainfall of Pasoh Forest Reserve (c) and Danum Valley Field Centre (d) over 13 years and 2 months. The vertical dotted line indicates 100 mm. (e, f) 30-day cumulative average rainfall at Pasoh Forest Reserve (e) and Danum Valley Field Centre (f). The horizontal dotted red line indicates 100 mm.
