# Supplementary material for: The genome of Shorea leprosula (Dipterocarpaceae) highlights the ecological relevance of drought in aseasonal tropical rainforests
Source: Commun Biol. 2021 Oct 7;4:1166. doi: 10.1038/s42003-021-02682-1 (PMC8497594; doi:10.1038/s42003-021-02682-1)
Supplement: Supplementary file 2 — Reporting summary. [file 42003_2021_2682_MOESM2_ESM.pdf]

nature research

Corresponding author(s): Kentaro Shimizu, Kevin Kit Siong Ng, Soon Leong Lee

Last updated by author(s): Aug 21, 2021

Reporting Summary

Nature Research wishes to improve the reproducibility of the work that we publish. This form provides structure for consistency and transparency in reporting. For further information on Nature Research policies, see our Editorial Policies and the Editorial Policy Checklist.

Statistics

For all statistical analyses, confirm that the following items are present in the figure legend, table legend, main text, or Methods section.

n/a	Confirmed	
☐	☒	The exact sample size (*n*) for each experimental group/condition, given as a discrete number and unit of measurement
☐	☒	A statement on whether measurements were taken from distinct samples or whether the same sample was measured repeatedly
☐	☒	The statistical test(s) used AND whether they are one- or two-sided<br>*Only common tests should be described solely by name; describe more complex techniques in the Methods section.*
☐	☒	A description of all covariates tested
☐	☒	A description of any assumptions or corrections, such as tests of normality and adjustment for multiple comparisons
☐	☒	A full description of the statistical parameters including central tendency (e.g. means) or other basic estimates (e.g. regression coefficient) AND variation (e.g. standard deviation) or associated estimates of uncertainty (e.g. confidence intervals)
☐	☒	For null hypothesis testing, the test statistic (e.g. *F*, *t*, *r*) with confidence intervals, effect sizes, degrees of freedom and *P* value noted<br>*Give P values as exact values whenever suitable.*
☐	☒	For Bayesian analysis, information on the choice of priors and Markov chain Monte Carlo settings
☒	☐	For hierarchical and complex designs, identification of the appropriate level for tests and full reporting of outcomes
☒	☐	Estimates of effect sizes (e.g. Cohen's *d*, Pearson's *r*), indicating how they were calculated

Our web collection on statistics for biologists contains articles on many of the points above.

Software and code

Policy information about availability of computer code

Data collection	*Provide a description of all commercial, open source and custom code used to collect the data in this study, specifying the version used OR state that no software was used.*
Data analysis	All related software (as cited in the manuscript): ALLPATHLGS v52488, K-mer Analysis Toolkit (KAT), Trinity v2.0.3, Trimmomatic v0.32, AUGUSTUS v3.2, BUSCO v3.1.0, RepeatMasker v4.0.5, RepeatModeler, MCScanX, VGCS v2.0, CODEML, PAML package, MAFFT version7, Gblocks version 0.9b, BEAST package v1.8, BWA v0.7.12, GATK v3.5, Picard 2.6.0, Beagle v4.1, libsequence package, VCFtools, PLINK v1.9, ADMIXTURE v1.3, topGO, MEGA7, SUSHI pipeline, STAR v2.4.2a & edgeR.

For manuscripts utilizing custom algorithms or software that are central to the research but not yet described in published literature, software must be made available to editors and reviewers. We strongly encourage code deposition in a community repository (e.g. GitHub). See the Nature Research guidelines for submitting code & software for further information.

Data

Policy information about availability of data

All manuscripts must include a data availability statement. This statement should provide the following information, where applicable:

- Accession codes, unique identifiers, or web links for publicly available datasets
- A list of figures that have associated raw data
- A description of any restrictions on data availability

Raw reads and genome assembly have been deposited to DDBJ as BioProject under accession numbers PRJDB8161 and PRJDB8182, respectively.

Field-specific reporting

Please select the one below that is the best fit for your research. If you are not sure, read the appropriate sections before making your selection.

☒ Life sciences ☐ Behavioural & social sciences ☐ Ecological, evolutionary & environmental sciences

For a reference copy of the document with all sections, see nature.com/documents/nr-reporting-summary-flat.pdf

Life sciences study design

All studies must disclose on these points even when the disclosure is negative.

Sample size	Our sample sizes and read depths enabled significance testing of genome assembly, annotation, resequencing and differentially-expressed genes and gene sets.
Data exclusions	No data was excluded.
Replication	All replication was successful.
Randomization	For transcriptome data used for gene prediction, samples were grouped based on their tissues/organ type.<br>For transcriptome data used for dry experiment, samples were grouped based on before and after treatment.
Blinding	No blinding was used, as no randomized controlled trials were conducted, and measurements were not prone to observer bias

Reporting for specific materials, systems and methods

We require information from authors about some types of materials, experimental systems and methods used in many studies. Here, indicate whether each material, system or method listed is relevant to your study. If you are not sure if a list item applies to your research, read the appropriate section before selecting a response.

Materials & experimental systems

n/a	Involved in the study	
☒	☐	Antibodies
☒	☐	Eukaryotic cell lines
☒	☐	Palaeontology and archaeology
☒	☐	Animals and other organisms
☒	☐	Human research participants
☒	☐	Clinical data
☒	☐	Dual use research of concern

Methods

n/a	Involved in the study	
☒	☐	ChIP-seq
☒	☐	Flow cytometry
☒	☐	MRI-based neuroimaging
